# Methionine Aminopeptidase 2 (MetAP2) Inhibitor BL6 Attenuates Inflammation in Cultured Microglia and in a Mouse Model of Alzheimer’s Disease

**DOI:** 10.3390/molecules30030620

**Published:** 2025-01-31

**Authors:** Xiuli Zhang, Shivakumar Subbanna, Colin R. O. Williams, Stefanie Canals-Baker, Audrey Hashim, Donald A. Wilson, Louis M. Weiss, Srushti Shukla, Parthiban Chokkalingam, Sasmita Das, Bhaskar C. Das, Mariko Saito

**Affiliations:** 1 Division of Neurochemistry, Nathan Kline Institute for Psychiatric Research, Orangeburg, NY 10962, USA; zhangxiuli20082015@gmail.com (X.Z.); subbanna.shivakumar@nki.rfmh.org (S.S.); scanalsbaker@gmail.com (S.C.-B.); drgah@aol.com (A.H.); 2 Emotional Brain Institute, Nathan Kline Institute for Psychiatric Research, Orangeburg, NY 10962, USA; colin.williams@nki.rfmh.org (C.R.O.W.); donald.wilson@nyulangone.org (D.A.W.); 3 Department of Child and Adolescent Psychiatry, New York University Medical Center, New York, NY 10016, USA; 4 Department of Pathology/Medicine, Albert Einstein College of Medicine, Bronx, NY 10461, USA; louis.weiss@einsteinmed.edu; 5 School of Pharmacy and Pharmaceutical Sciences, University at Buffalo, The State University of New York, Buffalo, NY 14201, USA; srushti.shukla@my.liu.edu (S.S.); parthibancnp@gmail.com (P.C.); sasmita.das@liu.edu (S.D.); 6 Department of Medicine, Icahn School of Medicine at Mount Sinai, New York, NY 10029, USA; 7 Department of Psychiatry, New York University School of Medicine, New York, NY 10016, USA

**Keywords:** MetAP2 inhibitors, microglia, neuroinflammation, SIM-A9, icv-STZ, Alzheimer’s disease, NF-κB, phospho-Akt, M1/M2 polarization

## Abstract

Methionine aminopeptidase 2 (MetAP2) plays an important role in the regulation of protein synthesis and post-translational processing. Preclinical/clinical applications of MetAP2 inhibitors for the treatment of various diseases have been explored because of their antiangiogenic, anticancer, antiobesity, antidiabetic, and immunosuppressive properties. However, the effects of MetAP2 inhibitors on CNS diseases are rarely examined despite the abundant presence of MetAP2 in the brain. Previously, we synthesized a novel boron-containing MetAP2 inhibitor, BL6, and found that it suppressed angiogenesis and adipogenesis yet improved glucose uptake. Here, we studied the anti-inflammatory effects of BL6 in SIM-A9 microglia and in a mouse model of Alzheimer’s disease generated by the intracerebroventricular (icv) injection of streptozotocin (STZ). We found that BL6 reduced proinflammatory molecules, such as nitric oxide, iNOS, IL-1β, and IL-6, together with phospho-Akt and phospho-NF-κB p65, which were elevated in lipopolysaccharide (LPS)-activated microglial SIM-A9 cells. However, the LPS-induced reduction in Arg-1 and CD206 was attenuated by BL6, suggesting that BL6 promotes microglial M1 to M2 polarization. BL6 also decreased glial activation along with a reduction in phospho-tau and an elevation in synaptophysin in the icv-STZ mouse model. Thus, our experiments demonstrate an anti-neuroinflammatory action of BL6, suggesting possible clinical applications of MetAP2 inhibitors for brain disorders in which neuroinflammation is involved.

## 1. Introduction

MetAPs are metallopeptidases that selectively catalyze the removal of the N-terminal methionine from newly synthesized proteins, playing a critical role in the regulation of post-translational processing and proper protein synthesis and functions [1,2]. There are two main MetAP isoforms in eukaryotes, MetAP1 and MetAP2 [3], that are essential for cellular growth and viability. While methionine aminopeptidase activity is shared by MetAP1 and MetAP2, each MetAP contains a unique N-terminal domain which links to a distinct site near the exit of the nascent polypeptide chains [4,5]. In MetAP2, its N-terminal polycharged domain is involved in association with eukaryotic initiation factor 2-α (eIF2α) and protects it from inhibitory phosphorylation, promoting general protein synthesis [3,6].

Since fumagillin, a natural product of fungal origin, was discovered as a potent inhibitor of angiogenesis [7], synthetic analogs of fumagillin such as AGM-1470 (TNP470) have been developed. Fumagillin and its analogs have been found to inhibit the methionine aminopeptidase activity of MetAP2, but not MetAP1, without affecting the ability of MetAP2 to block elF2α phosphorylation [8,9]. It has also been shown that fumagillin acts by covalently modifying His231 at the active site of MetAP2 [10]. While in early clinical studies, fumagillin analogs were tested primarily for oncologic indications and as an antiparasitic agent, studies to develop the clinical applications of these MetAP2 inhibitors have been expanded to other fields. MetAP2 inhibitors are not only antiangiogenic and anticancer but also show antiobesity and immunosuppressive properties [11]. New fumagillin analogs, such as beloranib and ZGN-1061, have been studied for their ability to improve obesity and glucose control [12,13,14]. Although the mechanism(s) of these effects of MetAP2 inhibitors have not been well elucidated, they may be partially due to the antiproliferative action of MetAP2 inhibitors against endothelial cells [8] and other sensitive cell types.

Previously, we reshaped the chemical structure of fumagillin to generate a boron atom containing the MetAP2 inhibitor BL6 and investigated the effect of BL6 on angiogenesis in human umbilical vein endothelial cells (HUVECs) and examined its antiadipogenic effect on 3T3-L1 pre-adipocytes during differentiation. It was found that BL6 suppressed angiogenesis in HUVECs and reduced adipogenesis in 3T3-L1 preadipocytes yet improved glucose uptake [15], suggesting the antidiabetic properties of BL6 as shown in studies using other MetAP2 inhibitors [16,17]. Thus, BL6 displays therapeutic potential for several diseases. While MetAP2 inhibitors generally alleviate multiple pathological conditions, the therapeutic potential of MetAP2 inhibitors in brain disorders has had limited studies despite the abundant presence of MetAP2 in the brain [18]. In the present study, we examined if BL6 affects inflammation in SIM-A9 microglial cells and in a mouse model of Alzheimer’s disease (AD). Neuroinflammation appears to play a primary role in AD pathogenesis by exacerbating Aβ burden and tau hyperphosphorylation [19,20], and it has been shown that MetAP2 inhibitors exert immunosuppressive properties [21], which have a strong correlation with antiangiogenic properties [22,23]. It has also been shown that Lodamin, a MetAP2 inhibitor, suppresses CD4+ T cell proliferation and reduces the production of Th1 and Th17, which release proinflammatory cytokines [24], and a fumagillin prodrug suppressed the inflammatory responses of macrophages activated by nitric oxide (NO) [25]. However, as far as we know, the effects of MetAP2 inhibitors on glial cells have not been examined. For the in vitro study, we used the SIM-A9 microglial cell line. For the in vivo study, we used a sporadic AD mouse model generated by an icv injection of STZ. Although the precise mechanisms are unknown, an icv injection of a low concentration of STZ induces an AD-like pathology, such as disturbances in brain insulin signaling and glucose utilization, accumulation of Aβ plaques, tau hyperphosphorylation, inflammation, and progressive cognitive impairments, in rodents [26,27,28,29,30]. Compared to transgenic mouse models, the icv-STZ model shows a more enhanced neuroinflammation, and the effects of anti-inflammatory agents have been often tested using the icv-STZ model [31,32].

Our studies indicated that BL6 reduced the proinflammatory responses of SIM-A9 microglial cells stimulated by LPS and decreased glial cell activation in an icv-STZ mouse model of AD. Since angiogenesis, inflammation, insulin resistance, and glucose hypometabolism can be ameliorated by BL6 and are involved in AD pathogenesis [33], BL6 may provide therapeutic opportunities for treating AD through its multi-target (antiangiogenic, antidiabetic, and anti-inflammatory) properties.

## 2. Results

### 2.1. BL6 Synthesis

**Figure 1:**
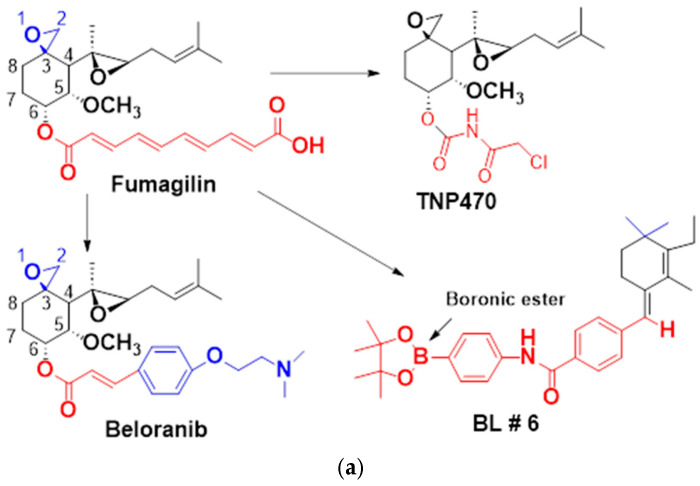

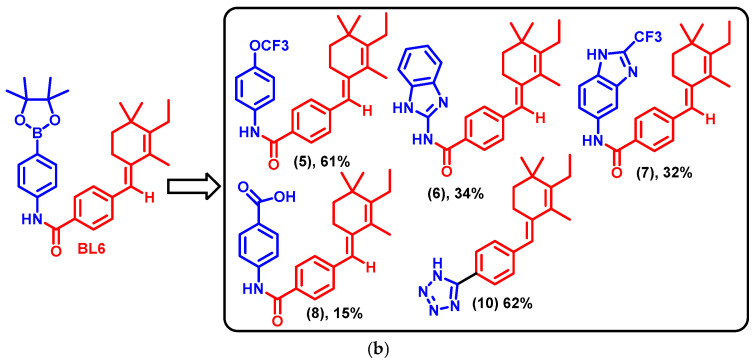
(**a**) Structure of BL6. Fumagillin was reshaped to generate a boron atom containing the MetAP2 inhibitor BL6 as described in the Materials and Methods. (**b**) SAR studies of BL6. BL6 was reshaped to generate more active compounds by substituting different amines to form amide bonds as described in the Supplementary Materials.

BL6 (Figure 1a) was synthesized by reshaping fumagillin to generate a boron atom containing MetAP2 inhibitor as described previously [15] and also briefly here in the Materials and Methods.

Our research began with the identification of BL6 as a promising active compound (Figure 1a). To explore its potential further, we conducted structure–activity relationship (SAR) studies. Based on the insights gained, we embarked on synthesizing various derivatives to enhance the compound’s activity. This led us to prepare different amide derivatives (Compounds **5**–**8**) and a tetrazole derivative (Compound **10**), each designed around the structure of BL6 (Figure 1b). Our primary goal is to develop derivatives of BL6 that exhibit enhanced biological activity. In our design approach, we focused on preserving the hydrophobic segment of BL6 while systematically modifying the hydrophilic part. This strategy is aimed at optimizing the compound’s interaction with biological targets, potentially leading to improved efficacy and potency. For detailed synthesis schemes and comprehensive analytical data, please refer to the Supplementary Materials accompanying this study. The synthesis procedures include the precise synthetic routes for each derivative, specifying the starting materials, reaction conditions, and purification methods employed. Analytical data such as NMR spectra, mass spectra, and purity assessments are provided to verify the identity and structural integrity of each synthesized compound.

### 2.2. Molecular Docking

The Cambridge Crystallographic Data Centre (CCDC) software (version 2023.1) GOLD was employed to perform docking studies to support the interaction and determine the preferred a binding model of the compound BL6. The active sites of the Human MetAP2 protein (PDB ID: 1b6a) and Protozoal MetAP2 (PDB ID: *3fmq*) were utilized for docking BL6. Discovery Studio Visualizer was used to generate 2D and 3D images of the docking and binding interactions. The crystallized structures of the proteins were derived, and the docking of the BL6 ligand was carried out using a series of progressively refined protocols available in GOLD. Initially, the virtual screening protocol was used to dock BL6 to both the entire surface of MetAP2 and specifically to the ligand binding site. Next, a rigid receptor protocol was employed to refine the docked conformations at the binding site. Finally, an induced fit protocol was used to allow for protein flexibility at the cleft binding site, further refining binding affinity.

**Figure 2:**
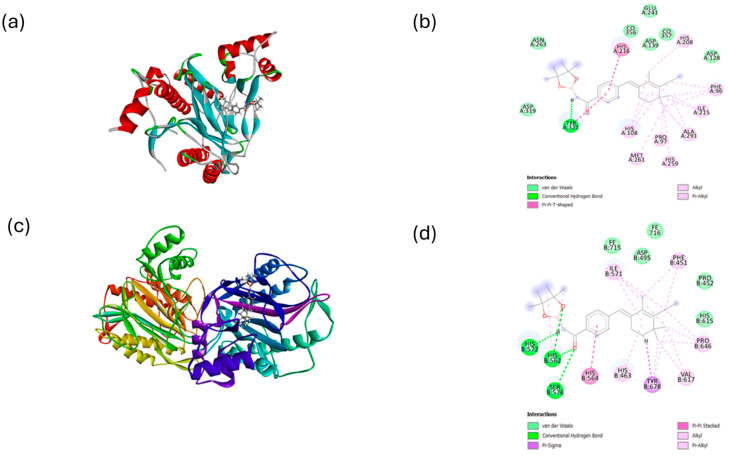
(**a**) 3D representation of HuMetAP2 (PDB ID:1b6a) with BL6. (**b**) Ligand interaction 2D mapping of HuMetAP2–BL-6 (**c**) 3D representation of EcMetAP2 (PDB ID:3fmq) with BL6. (**d**) Ligand interaction 2D mapping of EcMetAP2–BL-6.

BL6 exhibits a variety of molecular interactions with the amino acid residues lining the binding cavity and the di-metal co-factor CO^2+^ of human MetAP2 (Figure 2). One significant interaction is the hydrogen bond formed with the residue Tyr321, which plays a crucial role in stabilizing the ligand–protein complex and enhancing binding affinity. Additionally, BL6 engages in a pi–pi T-shaped interaction with His216, which involves the aromatic rings of both BL6 and the histidine residue, further contributing to the stability of the complex. Van der Waals interactions with the CO^2+^ co-factor and other residues in the binding cavity add to the binding affinity by providing additional non-covalent stabilization. Alkyl interactions with various residues also play a role in this stabilization process.

In contrast, BL6 exhibits stronger interactions with protozoal MetAP2. It forms hydrogen bonds with His572, His562, and Ser576, which are critical for strong binding and stability. Van der Waals interactions with Pro452, His615, Asp495, and the co-factor Fe(III) ion further enhance the binding affinity by providing a network of weak but collectively significant stabilizing forces. Additionally, BL6 participates in pi–sigma and pi–pi interactions with various residues, which contribute to the overall binding affinity observed with Protozoal MetAP2. These interactions illustrate the intricate ways in which BL6 can bind effectively to both human and protozoal MetAP2, highlighting the diverse types of molecular interactions involved.

### 2.3. ADMET Properties

We evaluated the physiochemical properties of BL6 using the Swiss ADME online platform. Table 1 summarizes not only all the physiochemical properties but the metabolism properties too. The in silico ADME and toxicity prediction was accomplished with the help of the PreADMET tool, which provides the latest and most inclusive information for the diverse chemicals associated with the known absorption, distribution, metabolism, excretion, and toxicity profiles. The ADME/toxicity properties are closely related to the physicochemical descriptors, such as lipophilicity (logP), molecular weight, polar surface area, and water solubility. The most well-recognized rule relating the chemical structures to their biological activities is Lipinski’s rule, called the rule of five. Several in vitro methods have been used in the drug selection process for assessing the intestinal absorption of drug candidates. Among them, the Caco-2 and MDCK (Madin Darby canine kidney) cell models have been recommended as reliable in vitro models for the prediction of oral drug absorption. For the absorption profiles, we have indicated F20% and F30%, which refer to the oral bioavailability of the drug or compound, indicating that 20–30% of the administered dose is absorbed into the bloodstream after oral administration. Meanwhile, human intestinal absorption (HIA) refers to the process and extent to which a drug or compound is absorbed through the human intestine following oral administration. In evaluating the distribution profile, we have mentioned plasma protein binding (PPB) and volume of distribution (VD), both pharmacokinetic parameters that describe the extent to which a drug distributes into the tissues and fluids of the body relative to its concentration in the blood or plasma, as well as blood–brain barrier (BBB) values. Regarding toxicity, we have included human hepatotoxicity (H-HT), which refers to the potential of a substance to cause liver damage in humans, AMES toxicity, and respiratory toxicity.

### 2.4. Effects of BL6 on the Cell Viability of SIM-A9 Cells Treated with a High Dose of LPS

**Figure 3:**
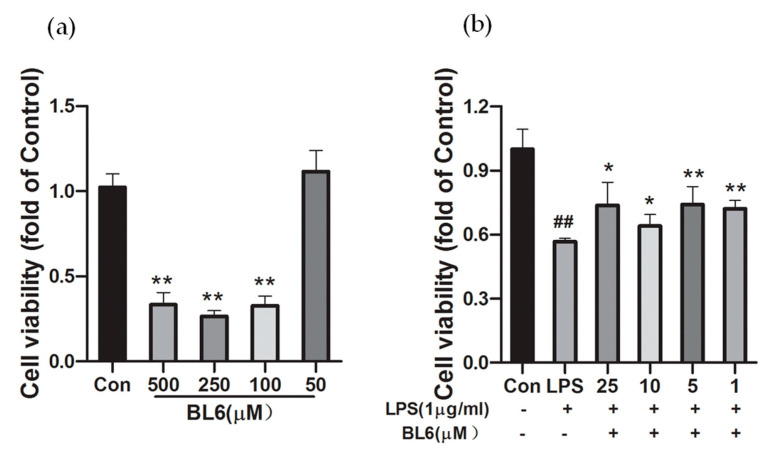
Effects of BL6 on the cell viability of SIM-A9 cells with or without LPS treatment. (**a**) Cell viability of SIM-A9 cells treated with BL6 for 24 h was assessed by an MTT assay. Results are expressed as the mean ± SD (*n* = 4) of fold changes to the control (Con). ** *p* < 0.01 compared with the control group. (**b**) SIM-A9 cells were pretreated with BL6 for 30 min, followed by treatment with LPS (1 μg/mL) for 24 h. Then, cell viability was assessed by an MTT assay. Results are expressed as the mean ± SD (*n* = 8). ## *p* < 0.01 compared with the control group, * *p* < 0.05, ** *p* < 0.01 compared with the LPS group.

The boron-containing MetAP2 inhibitor, BL6 (Figure 1), was synthesized as described previously [15]. SIM-A9 cells were incubated with 500, 250, 100, and 50 µM of BL6 for 24 h, and the cell viability was measured by an MTT assay (Figure 3a). BL6 was dissolved in DMSO followed by dilution in DMEM, and the final concentrations of DMSO were less than 0.1%. In Figure 3a, there are no statistically significant differences among the BL6 concentrations of 100, 250, and 500 μM. However, at a BL6 concentration of 50 μM, there is a significant statistical difference compared to the concentrations of 100, 250, and 500 μM. Since the results showed that 50 µM BL6 was not toxic to cells, we used concentrations lower than 50 µM for further experiments. In Figure 3b, SIM-A9 cells pretreated with BL6 (25, 10, 5, and 1 µM) for 30 min were also incubated with LPS (1 μg/mL) for 24 h, and cell viability was measured by an MTT assay. The results indicate that BL6 partially ameliorated the reduction in cell viability induced by a high dose (1 µg/mL) of LPS (Figure 3b). There are no statistically significant differences among the concentrations when measuring LPS-induced cell viability at BL6 concentrations of 1, 5, 10, and 25 μM.

### 2.5. Effects of BL6 on NO Production and Inflammatory Cytokines Induced by LPS in SIM-A9 Cells

**Figure 4:**
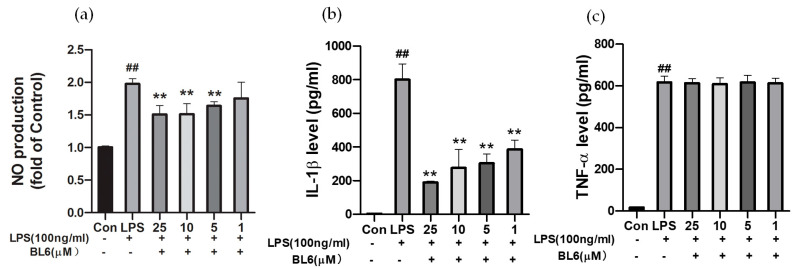
Effects of BL6 on NO production and inflammatory cytokines induced by LPS in SIM-A9 cells. (**a**) NO production of SIM-A9 cells pretreated with BL6 (25, 10, 5, and 1 µM) for 30 min followed by treatment with LPS (100 ng/mL) for 24 h was assessed using the Griess method. Results are expressed as the mean ± SD (*n* = 5) of fold changes to the control. ## *p* < 0.01 compared with the control group and ** *p* < 0.01 compared with the LPS group. (**b**) SIM-A9 cells were treated as described in (**a**) and the levels of IL-1β in the cell culture medium were measured via ELISA. Results are expressed as the mean ± SD (*n* = 4). ## *p* < 0.01 compared with the control group and ** *p* < 0.01 compared with the LPS group. (**c**) SIM-A9 cells were treated as described in (**a**), and levels of TNF-α in the cell culture medium were measured via ELISA. Results are expressed as the mean ± SD (*n* = 4). ## *p* < 0.01 compared with control group. The BL6 + LPS group under the concentrations of 1–25 µM did not show any significant differences compared with the LPS group.

SIM-A9 cells were pretreated with BL6 (25, 10, 5, and 1 µM) for 30 min, followed by treatment with LPS (100 ng/mL) for 24 h. While 1 µg/mL LPS was used to examine the protective effects of BL6 in the previous section (Figure 3b), 100 ng/mL LPS was chosen for activating SIM-A9 microglial cells because this concentration has been widely used for microglial activation in cultures and did not reduce cell viability. LPS increased NO production in the cell culture medium, and preincubation with BL6 reduced NO production significantly at concentrations of 5, 10, and 25 μM (Figure 4a). There were no statistically significant differences among the concentrations of 1, 5, 10, and 25 μM. IL-1β and TNF-α levels detected by ELISA in the cell culture medium also increased with the LPS treatment, and preincubation with BL6 significantly decreased IL-1β levels (Figure 4b). The BL6 concentration at 25 μM showed a statistically significant difference compared to the concentrations of 1 and 5 μM, while there were no statistically significant differences among the other concentrations. TNF-α levels were not reduced by preincubation with 1–25 µM BL6 (Figure 4c). Thus, BL6 decreased the proinflammatory factors (NO and IL-1β) that were enhanced by LPS, suggesting BL6 has anti-inflammatory effects.

### 2.6. BL6 May Promote M1 to M2 Microglial Polarization in SIM-A9 Cells Stimulated by LPS

**Figure 5:**
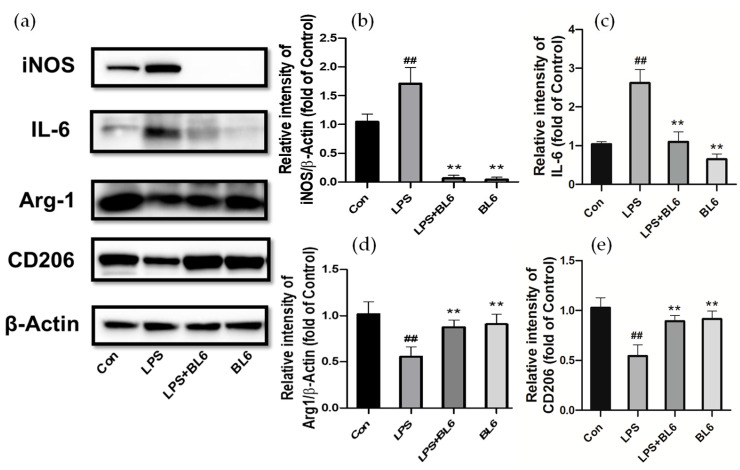
BL6 promotes M1 to M2 microglial polarization in SIM-A9 cells stimulated by LPS. SIM-A9 cells were pretreated with BL6 (10 µM) for 30 min before 100 ng/mL LPS treatment for 24 h, and iNOS, IL-6, Arg1, and CD206 expression in the cells was analyzed by Western blots. (**a**) Representative immunoblots for iNOS, IL-6, Arg1, CD206, and β-actin. (**b**–**e**) Quantitative analyses of immunoblot bands. iNOS (**b**), IL-6 (**c**), Arg1 (**d**), and CD206 (**e**) levels were normalized to β-actin. The data are expressed as the mean ± SD (*n* = 4). ## *p* < 0.01 compared with the control group, ** *p* < 0.01 compared with the LPS-treated group.

SIM-A9 cells pretreated with BL6 (10 µM) or the vehicle for 30 min were further incubated with 100 ng/mL LPS for 24 h, and iNOS, IL-6, Arg1, CD206, and β-actin expression in the cell lysates were analyzed by immunoblots. LPS was shown to increase the levels of iNOS and IL-6, while BL6 decreased these protein amounts (Figure 5a–c). Interestingly, BL6 alone totally inhibited iNOS expression in the control (Figure 5a,b). At the same time, we found that the levels of Arg1 and CD206 decreased in the LPS group, and BL6 attenuated this reduction (Figure 5a,d,e). Activated microglia are thought to differentiate into various phenotypes, including deleterious M1 and neuroprotective M2 phenotypes. “Classically activated” M1 microglia typically release damaging proinflammatory cytokines and reactive oxygen and nitrogen species that exacerbate brain damage, and the “alternatively activated” M2 phenotype secretes excess anti-inflammatory cytokines and trophic factors that resolve local inflammation and promote brain recovery [34,35,36,37]. Since phenotypic changes between M1 and M2 can occur under various conditions, balancing M1/M2 polarization is thought to be a therapeutic target for diseases caused or exacerbated by neuroinflammation [35]. In our experiment, BL6 decreased iNOS and IL-6, which are considered M1 markers, and increased Arg1 and CD206, which are considered M2 markers, indicating that BL6 promoted microglial M1 to M2 polarization.

### 2.7. Involvement of the PI3K/Akt/NF-κB Pathway in the Anti-Inflammatory Effects of BL6

**Figure 6:**
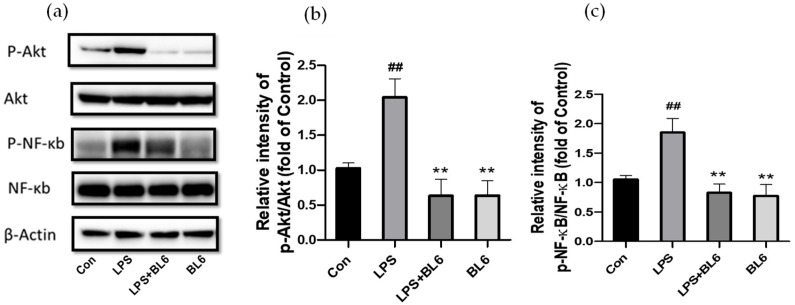
BL6 reduces phosphorylation of Akt and NF-κB p65 enhanced by LPS in SIM-A9 cells. Cells pretreated with BL6 (10 µM) for 30 min were incubated with 100 ng/mL LPS for 24 h, and cell homogenates were analyzed for ratios of p-Akt/Akt and p-NF-κB/NF-κB by Western blots. (**a**) Representative immunoblots for p-Akt, Akt, p-NF-κB, NF-κB, and β-actin. (**b**) Quantitative analysis of p-Akt/Akt ratios, presented as the fold of control. Protein expression levels were normalized to β-actin. The data are expressed as the mean ± SD (*n* = 4). ## *p* < 0.01 compared with the control group, ** *p* < 0.01 compared with the LPS group. (**c**) Quantitative analysis of p-NF-κB/NF-κB ratios, presented as the fold of control. Protein expression levels were normalized to β-actin. The data are expressed as the mean ± SD (*n* = 4). ## *p* < 0.01 compared with the control group, ** *p* < 0.01 compared with the LPS group.

**Figure 7:**
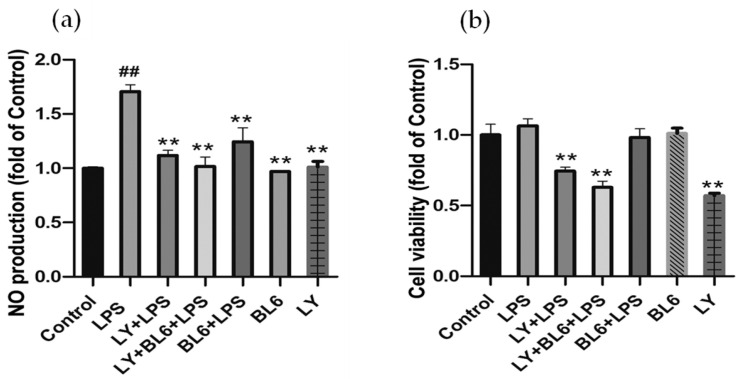
LY294002 inhibits NO formation by LPS in SIM-A9 cells. Cells were pretreated with/without LY294002 (10 µM) for 30 min and incubated with/without BL6 (10 µM) for 30 min before treatment with/without 100 ng/mL LPS for 24 h. Then, the cell viability and NO formation were determined. (**a**) NO production in the cell culture medium was assessed by the Griess method. The data are expressed as the mean ± SD (*n* = 4–8). ## *p* < 0.01 compared with the control group, ** *p* < 0.01 compared with the LPS-treated group. (**b**) Cell viability was assessed by an MTT assay. The data are expressed as the mean ± SD (*n* = 4–8). ** *p* < 0.01 compared with the LPS-treated group.

To explore the upstream pathways involved in the anti-inflammatory action of BL6 in SIM-A9 cells stimulated by LPS, the effects of BL6 on the activation of Akt and NF-κB were examined. NF-κB is a key nuclear transcription factor which mediates inflammatory responses [38,39], and the PI3K/Akt signaling pathway is considered an upstream regulator of NF-κB in LPS-treated microglia and other cell types [40,41,42,43,44,45,46]. SIM-A9 cells pretreated with BL6 (10 µM) or the vehicle for 30 min were cultured in the presence or absence of 100 ng/mL LPS for 24 h. Then, the expression of phospho-Akt (p-Akt), Akt, phospho-NF-κB p65 (p-NF-κB), NF-κB p65 (NF-κB), and β-actin in the cells was examined by immunoblots. Figure 6a demonstrates the representative images of the immunoblots, and Figure 6b,c presents the quantitative results of the ratios of p-Akt/Akt and p-NF-κB p65/NF-κB p65, respectively. The results indicated that the phosphorylation of Akt and NF-κB p65 increased in the LPS group compared with the control, and BL6 inhibited the elevation of p-Akt and p-NF-κB p65. Since phosphorylation of p65 (a subfamily of NF-κB proteins) at serine 536 enhances efficient transcriptional activation by NF-kB [47], elevation of p-NF-κB p65 indicates NF-κB activation. To examine the involvement of Akt phosphorylation in microglial activation, SIM-A9 cells were pretreated with/without LY294002 (10 µM), an inhibitor of phosphoinositide 3-kinase (PI3K) that phosphorylates Akt, for 30 min and incubated with/without BL6 (10 µM) for 30 min before treatment with/without 100 ng/mL LPS for 24 h. Then, cell viability and NO formation were determined by an MTT assay and the Griess reaction method. As shown in Figure 7a, pretreatment with LY294002 almost completely inhibited LPS-induced NO formation, although L294002 at this dose (10 µM) showed some reduction in cell viability or decreased cell proliferation (Figure 7b). These studies suggest that Akt phosphorylation is important for LPS-induced activation of microglia, and BL6 may exert anti-inflammatory action through inhibition of the Akt/NF-κB pathway.

### 2.8. BL6 Had No Protective Effect on STZ-Induced Neuro-2a Cytotoxicity

**Figure 8:**
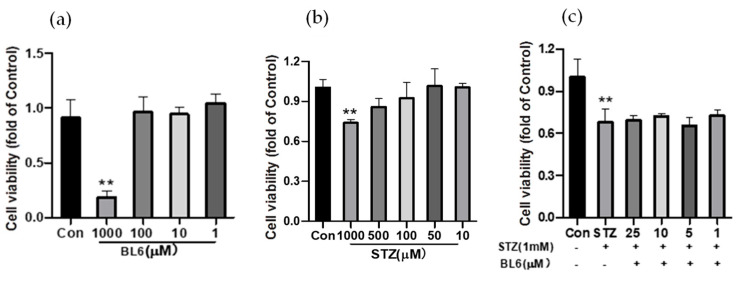
The effects of BL6 on Neuro-2a cell viability. (**a**) Cell viability of Neuro-2a cells treated with BL6 (1 mM, 100 µM, 10 µM, and 1 µM) for 24 h. (**b**) Cell viability of Neuro-2a cells treated with STZ (1 mM, 500 µM, 100 µM, 50 µM, and 10 µM) for 24 h. (**c**) Cell viability of Neuro-2a cells pretreated with BL6 (25, 10, 5, and 1 μM) for half an hour, followed by treatment with STZ (1 mM) for 24 h. Cell viability was assessed using an MTT assay. Results are expressed as the mean ± SD (*n* = 4). ** *p* < 0.01 compared with the control group.

To examine the effects of BL6 on neuronal cells, Neuro-2a cells were treated with BL6 (1 mM, 100 µM, 10 µM, and 1 µM) for 24 h, and cell viability was measured by an MTT assay. The results showed that concentrations of BL6 lower than 100 µM were not toxic to Neuro-2a cells (Figure 8a). Since in our in vivo study described below, we used a sporadic AD mouse model generated by an icv injection of STZ (icv-STZ model), we examined the effects of STZ on Neuro-2a cells. STZ (1 mM, 500 µM, 100 µM, 50 µM, and 10 µM) was administrated to Neuro-2a cells and incubated for 24 h to examine the cell damage. As shown in Figure 8b, the cell viability decreased in a dose-dependent manner, and 1 mM STZ inhibited cell viability to 74% of the control. Then, Neuro-2a cells were pretreated with BL6 (25, 10, 5, and 1 μM) for 30 min, followed by treatment with STZ (1 mM) for another 24 h, and the cell viability was determined by an MTT assay. The results showed that BL6 had no effect on the cell viability reduced by STZ, indicating that BL6 may not directly exert a protective effect on neurons injured by STZ (Figure 8c). Preliminary experiments also indicated that STZ (10 µM–1 mM) did not cause any significant reduction in the cell viability of SIM-A9 cells.

### 2.9. BL6 Reduced Icv-STZ-Induced Glial Activation in the Mouse Hippocampus

**Figure 9:**
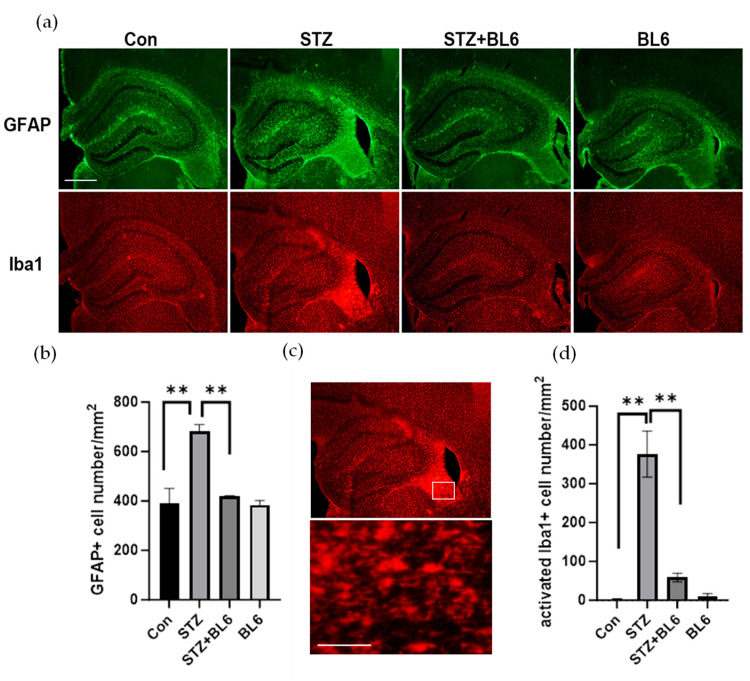
BL6 reduced icv-STZ-induced glial activation in the dorsal hippocampus. (**a**) GFAP+ astrocytes and Iba1+ microglia in the dorsal hippocampus regions of mice treated with icv-STZ (or vehicle) followed by BL6 (or vehicle) administration. The bar indicates 500 µm. (**b**) Densities of GFAP+ cells in the dorsal hippocampal fimbria. The data are expressed as the mean ± SEM (*n* = 3). ** *p* < 0.01. (**c**) The icv-STZ treatment increased rod- or amoeboid-like Iba1-positive cells with a large cell body in the fimbria. The lower panel is an enlarged image of the region shown as a square in the upper panel. The bar indicates 100 µm. (**d**) Densities of activated Iba1+ cells (rod- or amoeboid-like cells with a large cell body) in the dorsal hippocampal fimbria. The data are expressed as the mean ± SEM (*n* = 3). ** *p* < 0.001.

The effects of BL6 were also examined in vivo using an icv-STZ mouse model. Three days after 3-month-old C57BL/6 mice were treated with icv-STZ (or vehicle), BL6 (or vehicle) injections (once a day, 5 mg/kg, ip) started and continued for 3 weeks as described in the Materials and Methods. Then, mice were perfusion-fixed, and dual immunofluorescence staining of brain sections was performed for the detection of GFAP-positive (+) astrocytes and Iba1+ microglia in the dorsal hippocampus. In Figure 9a, a stronger GFAP labeling was apparent in the hippocampus of the STZ group compared to the other groups. Figure 9b shows that the densities of GFAP+ astrocytes in the fimbria augmented in icv-STZ mice were reduced by the BL6 treatment. Figure 9a also shows that STZ increased Iba1 labeling in the fimbria, while BL6 treatment decreased the intensity. Compared to the control group, BL6 alone had no effect on Iba1 labeling. In the fimbria, STZ increased rod- or amoeboid-like Iba1+ cells with a large cell body (Figure 9c). The quantitative measurement of the densities of those seemingly activated Iba1+ cells in the fimbria indicated that BL6 significantly reduced the activated microglial densities of the fimbria in icv-STZ mice (Figure 9d).

### 2.10. Effect of BL6 on GFAP, Synaptophysin, and p-Tau Levels in the Hippocampus of Icv-STZ-Treated Mice

**Figure 10:**
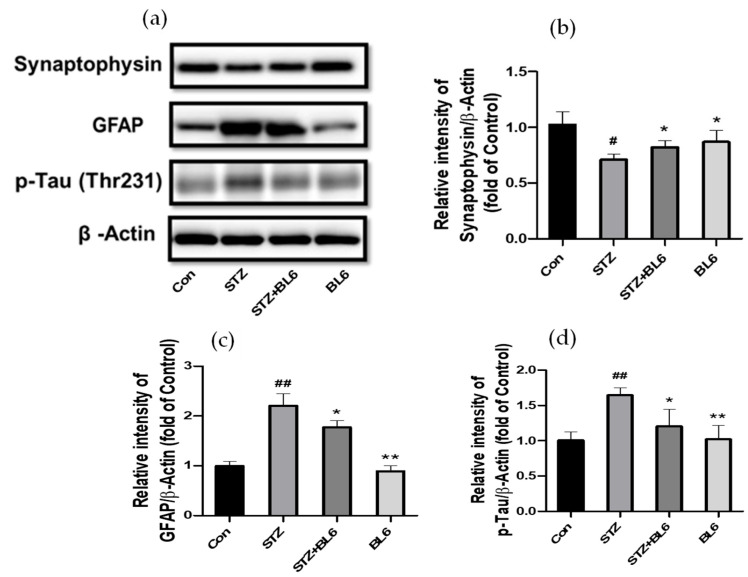
Effects of BL6 on synaptophysin, GFAP, and p-Tau expression in the hippocampus of icv-STZ-treated mice. The hippocampus of mice injected with 5 mg/kg of BL6 (or vehicle) for 3 weeks after icv-STZ injection was dissected for immunoblot analyses of synaptophysin, GFAP, and p-Tau. (**a**) Representative immunoblots. (**b**–**d**) Quantitative analyses of immunoblot bands for synaptophysin (**b**), GFAP (**c**), and p-Tau (**d**). The data are expressed as the mean ± SD (*n* = 4). ## *p* < 0.01, # *p* < 0.05 compared with the control group. ** *p* < 0.01, * *p* < 0.05 compared with the STZ-treated group.

Three-month-old C57BL/6 male mice were treated with icv-STZ (or the vehicle) and BL6 (or the vehicle) as described in the Materials and Methods, and the hippocampus was dissected out for analyses of synaptophysin, GFAP, p-Tau, and actin by immunoblots. The results indicated that BL6 reduced GFAP and p-Tau levels, which were increased in icv-STZ-treated mice (Figure 10a,c,d). In contrast, a reduction in the synaptophysin levels of STZ-treated mice was partially attenuated by the BL6 treatment (Figure 10a,b). Glial activation, p-Tau elevation, and decreases in synaptophysin are typical AD pathological characteristics and have been observed in icv-STZ-treated rodents [48,49,50,51]. Our results suggest that BL6 treatment reduced these characteristics observed in the hippocampus of icv-STZ-treated mice.

## 3. Discussion

Previously, we synthesized the small molecule BL6 (a boron-containing MetAP2 inhibitor) from fumagillin and reported its antiangiogenic, antiadipogenic, and antidiabetic properties [15]. Here, we have examined if BL6 has an anti-inflammatory ability as well because prior literature has indicated that MetAP2 inhibitors exert immunosuppressive properties [21,24,25]. Specifically, we aimed to examine the anti-inflammatory effects of BL6 on brain-resident immune cell microglia because the effects of MetAP2 inhibitors in the brain have rarely been examined, and to the best of our knowledge, the inhibitors’ effects on glia have not been reported before. Since neuroinflammation plays an important role in neurodegenerative diseases, including AD [19,20,52,53], the anti-neuroinflammatory property is a key target to explore for the clinical applicability of small molecules for neurodegenerative diseases.

We examined the anti-inflammatory effects of BL6 using a mouse microglial cell line, SIM-A9. We used SIM-A9 cells because this spontaneously immortalized cell line stably exhibits microglial phenotypes, including the expression of Iba1 and CD68, secretion of inflammatory mediators, and phagocytic activity, which are similar to cultured primary microglia [54,55]. Our studies using SIM-A9 cells indicated that BL6 generally induced anti-inflammatory properties in these cells. LPS increased iNOS levels and the resultant proinflammatory NO formation, both of which were reduced by BL6. Proinflammatory cytokines IL1-β and IL-6 increased by LPS were effectively reduced by BL6, although TNFα levels elevated by LPS were not significantly reduced by BL6 under our experimental conditions. Also, the increase in p-NF-κB P65 by LPS was reduced by BL6. Activation of NF-κB, a key nuclear transcription factor, is known to mediate inflammatory responses [38,39]. Activated NF-κB is translocated to the nucleus and is involved in the regulation of the transcription of many inflammation-related genes. Especially, the phosphorylation of p65 at serine 536 enhances efficient transcriptional activation by NF-kB [47]. Therefore, the elevation of phospho (ser536)-NF-κB p65 indicates NF-κB activation. Along with NF-κB activation, p-Akt was increased by LPS, and BL6 inhibited Akt phosphorylation. A PI3K inhibitor, LY294002, also reduced LPS-induced NO formation. These results agreed with the concept that the PI3K/Akt signaling pathway is an upstream regulator of NF-κB in LPS-treated microglia and other cell types, and PI3K inhibitors block the generation of proinflammatory mediators [40,41,42,43,44,45,46]. Activated Akt may function through IKK to promote NF-κB activation [42,46]. It has also been indicated that activation of the PI3K/Akt/mTOR pathway inhibits autophagy, which may induce neuroinflammation in microglia [56].

We also found that LPS decreased arginase 1 (Arg1) and CD206, and BL6 attenuated the reduction. Arg1 which converts arginine to polyamines, proline, and ornithine can effectively outcompete iNOS, which uses the same substrate arginine and downregulates the production of proinflammatory mediator NO. Thus, iNOS and Arg1 represent a set of markers for M1 and M2 microglia [34]. CD206, involved in the pinocytosis and phagocytosis of immune cells, has been shown to be positively associated with M2 polarization and play a role in regulating this phenotype [34,36]. Therefore, our results indicate that BL6 may induce M1 to M2 microglial polarization. However, M1/M2 polarization should be further examined using FACS or confocal microscopy in vitro and in vivo. It is possible that BL6 induces anti-inflammatory action (or M1 to M2 polarization) through inactivation of PI3K/Akt/NF-κB signaling, since both BL6 and LY29402 inhibit PI3K and induce anti-inflammatory activity in SIM-A9 cells treated with LPS, although we cannot exclude the possibility that BL6 induces anti-inflammatory action through some other mechanisms. Recently, we have reported the anti-inflammatory effects of retinoic acid receptor α (RARα) agonist, BT75, in SIM-A9 cells activated by LPS [57]. Compared to BT75, the anti-inflammatory effects of BL6 were generally more potent, and the reduction in p-Akt was much more robust. Since MetAP2 inhibitors can induce cell-type-dependent growth inhibition [58], BL6 may inhibit proliferation and reduce inflammation in SIM-A9 cells, as indicated in T cells treated with a MetAP2 inhibitor [24], although BL6 did not affect SIM-A9 viability/proliferation during 24 h of incubation with 100 ng/mL LPS (Figure 7). Compared to SIM-A9 cells, the cytotoxic effects of BL6 were much less in Neuro-2a cells (Figure 8). This may be due to the differences in the sensitivities of these cell lines against the antiproliferative action of MetAP2 inhibitors, although it has also been reported that Neuro-2a cells are rather resistant to the cytotoxic effects of certain toxins compared to other cell lines [59].

The icv-STZ AD mouse model was used to examine the anti-inflammatory effects of BL6 in vivo because this model produces pathological characteristics similar to sporadic AD, including neuroinflammation [26,27,28,29,30,31,32,51]. Our studies indicate that the icv-STZ treatment induced the activation of astrocytes and microglia in the hippocampus, especially in the fimbria, and injection of BL6 for 3 weeks attenuated the activation (Figure 9). Similar glial activation in the fimbria by icv-STZ treatment has been reported previously [60], and the authors speculate that the glial activation may be caused by STZ neurotoxicity to myelin. Interestingly, a recent literature review indicates that fornix degeneration is an early event in AD [61]. The reduction in GFAP in the hippocampus by BL6 detected by immunohistochemistry (Figure 9) agreed with the immunoblot results (Figure 10). In addition, BL6 decreased tau phosphorylation and attenuated synaptophysin reduction in the hippocampus of icv-STZ mice (Figure 10), suggesting partial alleviation of the AD pathology, although further biochemical, anatomical, and behavioral studies, including Aβ plaque analysis, using both male and female mice, will be necessary to evaluate the efficacy of BL6 to mitigate AD pathology and behavior.

The data reported in this paper indicate that in addition to the antiangiogenic and antidiabetic abilities previously reported [15], BL6 has anti-inflammatory activity in SIM-A9 microglia and in the icv-STZ mouse model of AD. One of the limitations of our current study is that we did not test other known MetAP2 inhibitors, such as fumagillin, together with BL6. Further studies may reveal that BL6 or other MetAP2 inhibitors may serve as a multi-target drug for AD.

## 4. Materials and Methods

### 4.1. Synthesis of BL6

BL6 was synthesized by reshaping the chemical structure of fumagillin as reported previously [15]. Final purification was achieved by silica gel column chromatography using ethyl acetate and hexane as eluents to obtain 0.112 g of compound BL6 as a white solid (23.0%, 0.23 mmol). Rf (ethyl acetate/n-hexane 1:5 *v*/*v*): 0.30. 1H NMR (400 MHz, DMSO-d6) δ 10.30 (s, 1H, -NH-), 7.93 (d, J = 8.41 Hz, 2H), 7.82 (d, J = 8.59 Hz, 2H), 7.65 (d, J = 8.54 Hz, 2H), 7.43 (d, J = 8.34 Hz, 2H), 6.49 (s, 1H), 2.58 (t, J = 6.24 Hz, 2H), 2.20 (q, J = 7.43 Hz, 2H), 1.88 (s, 3H), 1.46 (t, J = 6.24 Hz, 2H), 1.29 (s, 12H), 1.06 (s, 6H), 1.03 (t, J = 7.43 Hz, 3H). 13C NMR (400 MHz, DMSO-d6) δ 165.25, 147.97, 142.02, 141.68, 140.89, 134.99, 131.68, 128.83, 127.39, 126.53, 120.39, 119.10, 83.35, 38.10, 35.39, 27.29, 24.58, 22.26, 14.82, 14.54.

### 4.2. Cell Culture

The mouse microglial cell line SIM-A9 was obtained from ATCC (CRL3265, Manassas, VA, USA) and cultured in DMEM/F12 containing 10% fetal bovine serum, 5% horse serum, and 1% penicillin-streptomycin solution. The Neuro-2a cell line was also purchased from ATCC (CCL-131) and cultured in DMEM containing 10% fetal bovine serum and 1% penicillin-streptomycin solution. All cells were cultured under the conditions of 37 °C and 5% CO_2_ in an incubator.

BL6 (from 25 µM to 1 µM) used for the treatment of SEM-A9 cells was dissolved in DMSO followed by dilution in DMEM. The final concentrations of DMSO were less than 0.1%. For controls (0 µM BL6), the medium was used. We determined the doses of BL6 based on our previous studies [15] using human umbilical vein endothelial cells (HUVECs) and 3T3-L1 pre-adipocytes. Also, in SIM-A9 cells, we used doses of 25 µM or less, since 50 µM did not induce toxicity in these cells (Figure 3). The other reagents (LPS and STZ) used were dissolved in DMEM.

### 4.3. Preparation of the Icv-STZ Mouse Model and BL6 Administration

Three-month-old male C57BL/6 mice were purchased from Jackson Laboratory (Bar Harbor, ME, USA). All procedures described below were approved by the Nathan Kline Institute IACUC and were in accordance with the NIH guidelines for the proper treatment of animals. The mice were injected (icv) with STZ at 3 mg/kg body weight to induce an AD-like model using a slightly modified version of previously reported methods [29,30,31]. The scalp of the mouse was incised to locate the bregma. Small burr holes (1 mm in diameter) were drilled, and STZ in citrate buffer (50 mM, pH 4.5) (volume was calculated according to the mouse weight) was injected into both sides of the lateral ventricle with a microsyringe fitted with a 26-gauge blunt cannula at a rate of 0.5 μL/min with the following coordinates: 0.2 mm posterior to the bregma, ±1.0 mm lateral to the sagittal line, and 2.5 mm ventral to the surface of the skull. The cannula was withdrawn after remaining for 5 min in the injection position. The incision was closed with wound clips, and the animal was allowed to recover under a heat lamp. Sham-operated mice had received the same surgical procedures except with an injection of the same volume of the vehicle (50 mM citrate buffer).

**Figure 11:**
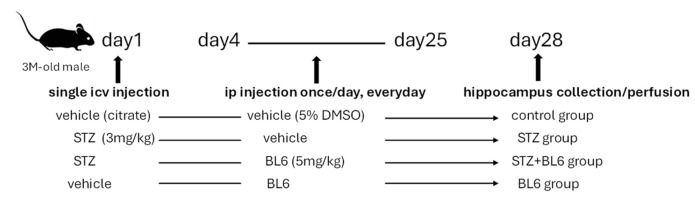
Preparation of the icv-STZ mouse model and BL6 administration.

As illustrated in Figure 11, three days after the icv-STZ injection, BL6 (in 5% DMSO in saline, 5 mg/kg body weight, STZ + BL6 group) or the vehicle (5% DMSO in saline) (STZ group) was given to the mice via intraperitoneal (ip) injection once a day for 3 weeks. Sham-operated mice were also given the vehicle (control group) or BL6 (5 mg/kg body weight, BL6 group). The dose of BL6 was based on previous literature using MetAP2 inhibitors in mouse studies [62,63]. After drug administration, the hippocampus was collected for immunoblot assays, or the brain was perfusion-fixed for immunofluorescence staining.

### 4.4. Cell Viability Detection

The MTT method was used for cell viability detection. SIM-A9 cells (2 × 10^5^ cells/well) were propagated in 96-well plates. SIM-A9 cells were treated with different doses (500, 250, 100, and 50 µM) of BL6 for 24 h or pretreated with different doses of BL6 (25, 10, 5, and 1 µM) for 30 min and then subjected to 24 h incubation with 1 µg/mL LPS. MTT was added to the cell culture with a final concentration of 0.5 mg/mL. After 4 h incubation, the cell medium was removed, and 100 µL DMSO was added to dissolve the crystal. The absorbance was measured at 570 nm with a reference wave at 630 nm. Neuro-2a cells were treated with BL6 (1 mM, 100 µM, 10 µM, and 1 µM) or STZ (1 mM, 500 µM, 100, 50 µM, 10 µM, and 1 µM) for 24 h, and cell viability was detected by MTT. Also, the cell viability of Neuro-2a cells pretreated with BL6 (25, 10, 5, and 1 μM) for 30 min followed by treatment with STZ (1 mM) for another 24 h was assessed using the MTT assay.

### 4.5. Nitric Oxide (NO) Detection

The Griess reaction method was used for the detection of NO production. Due to its extreme instability, NO is quickly and proportionally metabolized to nitrite. Therefore, nitrite has been extensively used as an indicator of NO. SIM-A9 cells (2 × 10^5^ per well) were cultured in 96-well plates overnight. Then, the cells were subjected to 30 min incubation of BL6 (25, 10, 5, and 1 µM), followed by 24 h stimulation with 100 ng/mL LPS. The same volume of the cell culture medium and Griess agent solution were mixed together, and the absorbance at 540 nm was detected after 30 min.

### 4.6. Inflammatory Cytokine Detection

An enzyme-linked immunosorbent assay (ELISA) was used to detect inflammatory factors in the cell culture medium. SIM-A9 cells (2 × 10^5^ per well) were cultured in 96-well plates overnight. Then, the cells were pretreated with BL6 (25, 10, 5, and 1 µM) for 30 min, followed by treatment with LPS (100ng/mL) for another 24 h. TNF-α and IL-1β in the cell culture medium were detected by the commercial ELISA kits TNFα Quantikine ELISA kit (R&D Systems, Minneapolis, MN, USA, Cat# MTA00B) and IL-1β mouse ELISA kit (Thermo Fisher Scientific, Watham, MA, USA, Cat# BMS6002), respectively, according to the manufacturers’ instructions.

### 4.7. Immunoblot

Immunoblot assays were performed using both SIM-A9 cells and brain hippocampus tissue as previously described [57]. Cells were pretreated with BL6 (10 µM) for 30 min before 100 ng LPS treatment for 24 h, then the cells were collected and lysed with RIPA lysis buffer plus protease and phosphatase inhibitors to detect Akt, phospho (p)-Akt, NF-κB p65, p-NF-κB p65, iNOS, Arginase 1 (Arg1), IL-6, CD206, and β-actin expression by Western blot. The hippocampus was also lysed and nNOS, GFAP, synaptophysin, p-Tau, and β-actin expression were detected by Western blot. Briefly, total protein amounts were measured by a Pierce BCA protein assay, and a 20 µg amount of total protein from each sample was separated by SDS-PAGE gel and transferred to nylon membranes. After blocking with 5% non-fat milk powder in TBST for 2 h at room temperature, these membranes were incubated with primary antibodies against Akt (Cat#4685), p-Akt (Cat#4060), NF-κB p65 (Cat#8242), p-NF-κB p65 (Cat#8242), iNOS (Cat#13120), Arg1 (Cat#93668), β-actin (Cat#3700), nNOS (Cat#4231), GFAP (Cat#3670), p-Tau (p-Thr231, Cat#71429), synaptophysin (Cat#36406), CD206/MRC1 (Ca#24595), and IL-6 (Cat#12912) [all from Cell Signaling Technology (Danvers, MA, USA) with a dilution of 1:1000] and corresponding secondary antibodies [Millipore Sigma (Burlington, MA, USA) with a dilution of 1:2500]. The bands were shown with an enhanced chemiluminescence (ECL) method and analyzed by Image J software version 1.53e.

### 4.8. Immunofluorescence Staining

Immunofluorescence staining was performed as previously described [64]. Mice were perfused with a solution containing 4% paraformaldehyde and 4% sucrose in cacodylate buffer (pH 7.2), and their heads were removed and further fixed in the perfusion solution overnight. Then, brains were removed, transferred to phosphate-buffered saline (PBS) solution, and kept at 4 °C for 2–5 days until cut with a vibratome into 50 µm thick coronal sections. The free-floating sections were rinsed in PBS, permeabilized in methanol for 10 min, and incubated for 30 min in blocking solution (PBS containing 5% BSA and 0.1% Triton X-100), followed by incubation overnight with antibodies against GFAP (GA5) (mouse mAb Cat#3670, Cell Signaling, with a dilution of 1:500) and Iba1 (rabbit polyclonal Cat#019-19741, FujifilmWako Chemicals, Richmond, VA, USA, with a dilution of 1:500), rinsed in PBS three times, followed by incubation with Alexa Fluoro594 goat anti-rabbit IgG and Alexa Fluoro488 goat anti-mouse IgG (Life Technologies, Grand Island, NY, USA) in 0.1% Triton X-100 in PBS containing 1% BSA for 1 h at r.t. Sections were finally rinsed in PBS three times, mounted, and coverslipped using ProLong Gold Antifade Reagent (Life Technologies). Glial activation was evaluated in the dorsal hippocampus region, especially in the fimbria, where activation was prominent. For quantitative measurement, the GFAP-positive cell number or rod- or amoeboid-like Iba1-positive cell number was counted in the hippocampal fimbria using 3 sections (around the bregma −1.34 to −1.82) from each brain, and 3 brains per each treatment group were analyzed. Cell counting and the measurement of area of interest (AOI) were performed by Image J software.

### 4.9. Statistical Analysis

Data were presented in the form of the mean ± SD or mean ± SEM. A one-way analysis of variance (ANOVA) with a Tukey test was performed to analyze the data using GraphPad Prism (version 9.5.1) or SPSS (version 24) software, and *p* < 0.05 was defined as statistically significant.

### 4.10. Molecular Docking and ADMET Profiling

Molecular docking was conducted utilizing CCDC GOLD employing a genetic algorithm, with subsequent generation of 2D and 3D representations using Discovery Studio (BIOVIA). ADMET profiling was predicted through ADMETlab2.0. All parameters in Table 1 are derived through in silico study, SMILES strings, and SDF/TXT/CSV formatted files uploaded to the software to derive the parameter. ADMETLab 3.0 predicts compounds’ ADMET (absorption, distribution, metabolism, excretion, and toxicity) properties using machine learning models and molecular data. It analyzes the chemical structure of a compound and calculates its molecular descriptors (such as size, shape, and functional groups). These descriptors are then fed into trained models to predict how the compound will behave in the body.

## Figures and Tables

**Table 1:** Physiochemical and ADMET properties of BL6.

Property	Characteristics	Value
**Physicochemical Properties**	Molecular Weight	409.28
	TPSA	47.56
	logS (Aqueous Solubility)	−4.973 log mol/L (Poor)
	logP	5.713 log mol/L
	logD	4.263 log mol/L
**Medicinal Chemistry**	Lipinski Rule	Accepted
**Absorption**	Caco-2 Permeability **	−4.809 cm/s (Excellent)
	HIA *	0.008 (Excellent)
	F20% *	0.006 (Excellent)
	F30% *	0.043 (Excellent)
**Distribution**	PPB (%)	98.37 (Poor)
	VD (L/kg)	2.888 (Excellent)
	BBB penetration *	0.018 cm/s (Excellent)
**Metabolism** **(CYP450 Inhibition)**	CYP1A2 Inhibitor *	0.251 (Active)
	CYP2C19 Inhibitor *	0.764 (Active)
	CYP2C9 Inhibitor *	0.771 (Active)
	CYP2D6 Inhibitor *	0.25 (Active)
	CYP3A4 Inhibitor *	0.137 (Active)
**Excretion**	CL (mL/min/kg)	3.382
	t_1/2_ (hr) ***	0.152
**Toxicity**	hERG Blockers *	0.019 µM (Excellent)
	H-HT *	0.79 (Toxic)
	AMES Toxicity *	0.543 (Moderately toxic)
	Respiratory Toxicity *	0.019 (Non-Toxic)

* The output value is the probability of the undesirable effect happening, with a number approaching the value of 1 being an indication of the certainty of the event taking place. ** Optimal: higher than −5.15 Log unit. *** Category 1: long half-life; Category 0: short half-life. Long half-life: >3 h; short half-life: <3 h. The output value is the probability of having a long half-life.

## Data Availability

All data are available in the article or upon request to the corresponding authors.

## Supplementary Materials

The following supporting information can be downloaded at: https://www.mdpi.com/article/10.3390/molecules30030620/s1, Figure S1: 1H & 13C NMR data of Compound **4**; Figure S2: 1H, 13C & 19F NMR data of Compound **5**; Figure S3: LCMS data of Compound **5**; Figure S4: 1H & 13C NMR data of Compound **6**; Figure S5: LCMS data of compound **6**; Figure S6: 1H, 13C & 19F NMR data of Compound **7**; Figure S7: LCMS data of Compound **7**; Figure S8: 1H & 13C NMR data of Compound **8**; Figure S9: LCMS data of Compound **8**; Figure S10: 1H & 13C NMR data of Compound **9**; Figure S11: LCMS data of Compound **9**; Figure S12: 1H & 13C NMR data of Compound **10**; Figure S13: LCMS data of Compound **10**; Figure S14: (A) 3D representation of HuMetAP2 (PDB ID:1b6a) with BL6; (B) Ligand interaction 2D mapping of HuMetAP2/BL-6; (C) 3D representation of EcMetAP2 (PDB ID:3fmq) with BL6. (D) Ligand interaction 2D mapping of EcMetAP2/BL6; Figure S15: Original uncropped immunoblot images; Scheme S1: Synthetic route for Compounds **5**–**8**; Scheme S2: Synthetic route for Compounds **10**.
