# Supplementary material for: Methionine Aminopeptidase 2 (MetAP2) Inhibitor BL6 Attenuates Inflammation in Cultured Microglia and in a Mouse Model of Alzheimer’s Disease
Source: Molecules. 2025 Jan 31;30(3):620. doi: 10.3390/molecules30030620 (PMC11820257; doi:10.3390/molecules30030620)
Supplement: Supplementary file 1 [file molecules-30-00620-s001.zip › molecules-3281279-SI.pdf]

Methionine Aminopeptidase 2 (MetAP2) Inhibitor BL6 Attenuates Inflammation in Cultured Microglia and in a Mouse Model of Alzheimer's Disease

Xiuli Zhang [1], Shivakumar Subbanna [1], Colin R. O. Williams [2], Stefanie Canals-Baker [1], Audrey Hashim [1], Donald A. Wilson [2,3], Louis M. Weiss [4], Srushti Shukla [5], Parthiban Chokkalingam [5], Sasmita Das [5], Bhaskar C. Das [5,6,*] and Mariko Saito [1,7,*]

[1] Division of Neurochemistry, Nathan Kline Institute for Psychiatric Research, Orangeburg, NY 10962, USA; zhangxiuli20082015@gmail.com (X.Z.); subbanna.shivakumar@nki.rfmh.org (S.S.); scanalsbaker@gmail.com (S.C.-B.); drgah@aol.com (A.H.)
[2] Emotional Brain Institute, Nathan Kline Institute for Psychiatric Research, Orangeburg, NY 10962, USA; colin.williams@nki.rfmh.org (C.R.O.W.); Donald.Wilson@nyulangone.org (D.A.W.)
[3] Department of Child and Adolescent Psychiatry, New York University Medical Center, New York, NY 10016, USA
[4] Department of Pathology/Medicine, Albert Einstein College of Medicine, Bronx, NY 10461 USA; louis.weiss@einsteinmed.edu
[5] School of Pharmacy and Pharmaceutical Sciences, University at Buffalo, The State University of New York, Buffalo, NY, USA; srushti.shukla@my.liu.edu (S.S.); parthibancnp@gmail.com (P.C.); sasmita.das@liu.edu (S.D.)
[6] Department of Medicine, Icahn School of Medicine at Mount Sinai, New York, NY 10029, USA
[7] Department of Psychiatry, New York University School of Medicine, New York, NY 10016, USA
* Correspondence: bhaskard@buffalo.edu (B.C.D.); mariko.saito@nki.rfmh.org (M.S.)

Table of contents

Description	Page No
1.0 General Information	**2**
2.0 Chemical Synthetic Schemes of compounds 5, 6, 7, 8, and 10	**2-3**
3.0 Synthetic procedure and their spectral details	**3-8**
4.0 Spectra of the title compounds	**9-24**
5.0 Molecular Docking	**25-26**

1.0 General Information

Compounds used as starting materials and reagents were obtained from SIGMA-Aldrich, ACROS ORGANICS, FISHER SCIENTIFICS, or other chemical companies, and utilized without further purification. Thin-layer chromatography (TLC) and column chromatography (CC) were performed with Kieselgel 60 F254 (Merck) and silica gel (Kieselgel 60, 230–400 mesh, Merck), respectively. Since all the compounds prepared contain aromatic rings, they were visualized and detected on TLC plates with UV light (short-wave, long-wave, or both). NMR spectra were recorded on a BRUKER AVANCE NEO NANOBAY-USA (400 MHz for 1H NMR, 62.5 MHz for 13C NMR, and 376.5 MHz for 19F NMR), and chemical shifts were calibrated to TMS (tetramethylsilane). All 19F NMR chemical shifts were referenced to external CF3CO2H (0.0 ppm). Chemical shifts (δ) were recorded in ppm and coupling constants (J) in hertz (Hz). Signal patterns are indicated as s, singlet; d, doublet; dd, doublet of doublets; t, triplet; m, multiplet and bs, broad singlet.

2.0 Chemical Synthetic Schemes of compounds 5,6,7,8, and 10

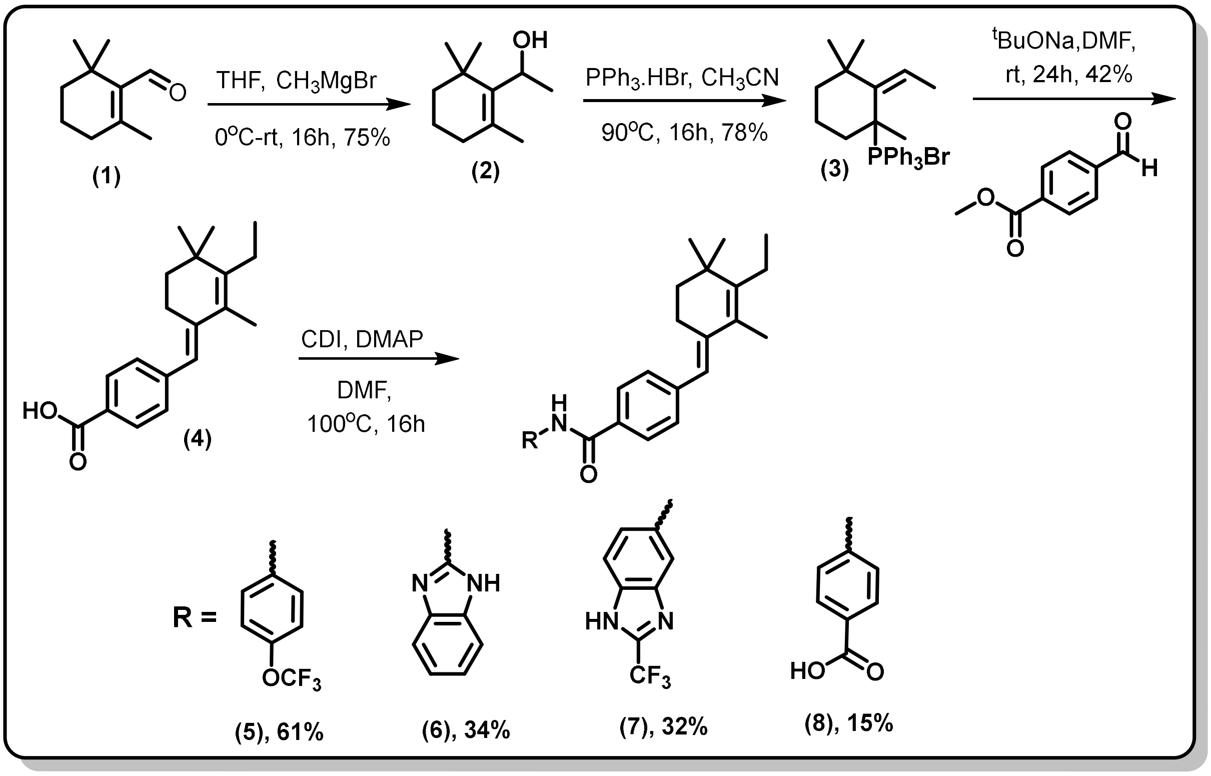

Scheme S1. Synthetic route for compounds **5-8**

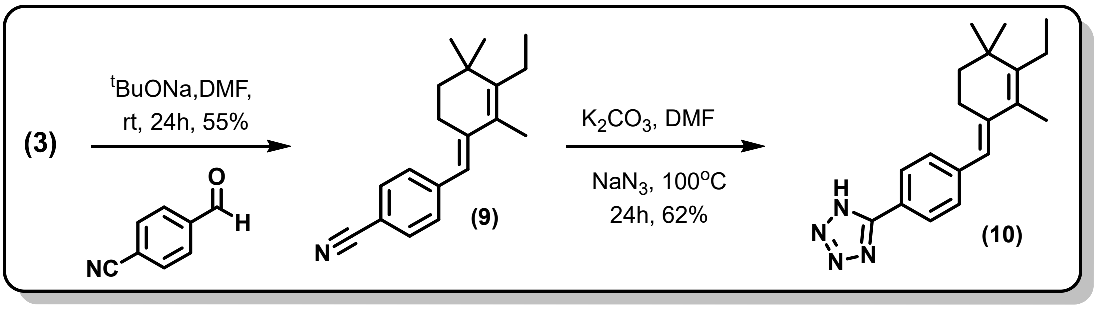

Scheme S2. Synthetic route for compounds **10**

3.0 Synthetic procedure for 5-10:

Synthesis of 1-(2,6,6-trimethylcyclohex-1-en-1-yl)ethan-1-ol (2):

A clean oven-dried three-necked round bottom flask was charged with a magnet, the addition of aldehyde (1 equiv.) (**1**), and dry THF at 0 °C under a nitrogen atmosphere. Slowly add the methyl magnesium bromide (3 equiv.) (Solution in THF) to the aldehyde solution and stir the reaction mixture for 30 minutes at 0°C. Then, the reaction mixture was allowed to be at room temperature by removing the ice-water bath and stirred for 16 h. Progress of the reaction was monitored by TLC, by looking at the disappearance of aldehyde, the polar spot observed with respect to aldehyde which was confirmed by the 2,4-DNP test (product is UV inactive). After completion of the reaction, the reaction mixture was quenched with a saturated aqueous solution of ammonium chloride (NH_4Cl). After neutralization, ethyl acetate was added to the reaction mixture; the combined organic layer was collected and dried with Na_2SO_4 and concentrated under reduced pressure. Alcohol (**2**) will be used for the next step without any further purification.

Synthesis of Wittig salt (3)

A clean oven-dried 50 mL round bottom flask was charged with **2** (1 equiv.) in acetonitrile (10 mL), then added by PPh_3.HBr (1 equiv.) and the resulting reaction mixture were stirred at 90^0C for 16 hr. Progress of the reaction was monitored by TLC (100 % Ethyl acetate). In the TLC plate polar spot was observed which indicates the salt formation. After completion of the reaction, the reaction mass was allowed to cool at ambient temperature. Acetonitrile was evaporated on

rotavapor, and the product was purified by recrystallization technique (Hexane). After recrystallization white solid (**3**) was observed. The white solid will be used for the next step.

Synthesis of (E)-4-((3-ethyl-2,4,4-trimethylcyclohex-2-en-1-ylidene)methyl)benzoic acid (4)

A clean oven-dried two-neck round bottom flask (RBF) was charged with salt (**3**) (1 equiv.) and methyl 4-formyl benzoate (1 equiv.) in DMF and the reaction mixture was stirred at room temperature until the formation of clear solution (10 minutes). Sodium tertbutoxide (3 equiv.) was added to the reaction mixture at 0 ^{0}C under a nitrogen atmosphere portion-wise. The reaction mixture was stirred at room temperature for 24 hr. Progress of the reaction was monitored by TLC (50 % Hexane: 50 % ethyl acetate), In TLC, polar spot was observed corresponding to aldehyde (4). After completion of the reaction, the reaction mixture was neutralized by 2N HCl. After neutralization, ethyl acetate was added to the reaction mixture; the combined organic layer was collected and dried with Na_2SO_4 and evaporated under reduced pressure. The crude material was purified by column chromatography.

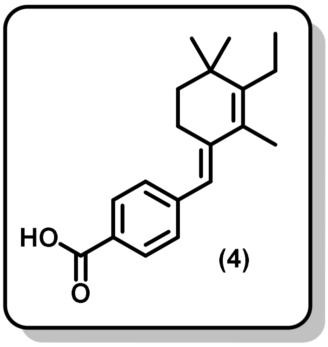

Pale yellow solid (42%)

^{1}H NMR (400 MHz, DMSO-d_6) δ 12.82 (s, 1H), 7.89 (d, J = 8.0 Hz, 2H), 7.40 (d, J = 8.1 Hz, 2H), 6.48 (s, 1H), 2.57 (d, J = 6.4 Hz, 2H), 2.20 (q, J = 7.5 Hz, 2H), 1.87 (s, 3H), 1.46 (d, J = 4.4 Hz, 2H), 1.05 (d, J = 11.9 Hz, 9H). ^{13}C NMR (101 MHz, DMSO-d_6) δ 167.68, 148.78, 143.39, 141.78, 129.60, 129.55, 128.33, 127.12, 121.04, 38.67, 35.96, 27.84, 24.30, 22.85, 15.38, 15.10.

General procedure for the synthesis of compounds 5-8:

An oven-dried 10 mL round bottom flask was charged with acid (1 equiv.) and CDI (1.5 equiv.) in DMF (3 mL). The reaction mixture was stirred at 70 °C for 30 minutes under a nitrogen atmosphere, then added by substituted amines (1.5 equiv.) and 4-dimethylaminopyridine (DMAP)

(2 equiv.), and the resultant mixture was stirred at 90 °C for 16 h. Reaction progress was monitored by TLC. After completion of the reaction, the reaction mass was allowed to cool at ambient temperature, diluted with water (10 mL), and extracted with EtOAc (3 × 10 mL). The combined organic layer was dried with anhydrous Na_2SO_4 and evaporated under reduced pressure. The crude material was purified by column chromatography.

(E)-4-((3-ethyl-2,4,4-trimethylcyclohex-2-en-1-ylidene)methyl)-N-(4-(trifluoromethoxy)phenyl)benzamide (5):

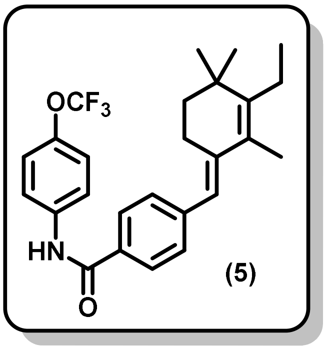

White solid (Yield = 61 %)

^{1}H NMR (400 MHz, DMSO-d_6) δ 10.38 (s, 1H), 7.94 – 7.89 (m, 4H), 7.41 (d, 8.3 Hz, 4H), 6.50 (s, 1H), 2.59 (s, 2H), 2.21 (d, J = 7.3 Hz, 2H), 1.88 (s, 3H), 1.46 (s, 2H), 1.07 –1.02 (m, 9H).^{13}C NMR (101 MHz, DMSO-d_6) δ 165.84, 148.61, 142.37, 141.53, 138.96, 132.10, 129.45, 127.97, 127.13, 122.08, 121.93, 121.05, 38.70, 35.99, 27.88, 24.28, 22.85, 15.40, 15.13. ^{19}F NMR (376.5 MHz, DMSO-d_6) δ -56.9. MS (ESI) calcd for $C_{26}H_{29}F_3NO_2$ 444.2145 found 444.2317 $[M+H]^+$.

(E)-N-(1H-benzo[d]imidazol-2-yl)-4-((3-ethyl-2,4,4-trimethylcyclohex-2-en-1-ylidene)methyl)benzamide (6):

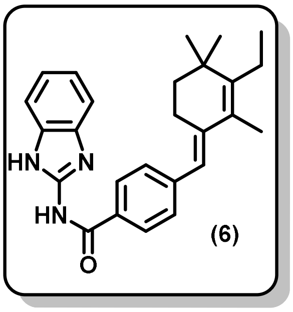

Pale yellow solid (Yield = 42%)

^{1}H NMR (400 MHz, DMSO-d_6) δ 12.21 (s, 2H), 8.11 (d, J = 7.9 Hz, 2H), 7.52 – 7.36 (m, 4H), 7.18 – 7.10 (m, 2H), 6.51 (s, 1H), 2.60 (t, J = 6.3 Hz, 2H), 2.21 (q, J = 7.5 Hz, 2H), 1.89 (s, 3H), 1.47 (t, J = 6.3 Hz, 2H), 1.06 (d, J = 12.8 Hz, 9H). ^{13}C NMR (101 MHz, DMSO-d_6) δ 168.28,

149.24, 148.63, 142.68, 141.61, 131.76, 129.44, 129.29, 128.56, 127.17, 121.91, 121.14, 38.71, 35.97, 27.86, 24.34, 22.86, 15.40, 15.12. MS (ESI) calcd for $C_{26}H_{29}N_3O$ 399.5380 found 399.9096 [M+H].

(E)-4-((3-ethyl-2,4,4-trimethylcyclohex-2-en-1-ylidene)methyl)-N-(2-(trifluoromethyl)-1H-benzo[d]imidazol-5-yl)benzamide (7):

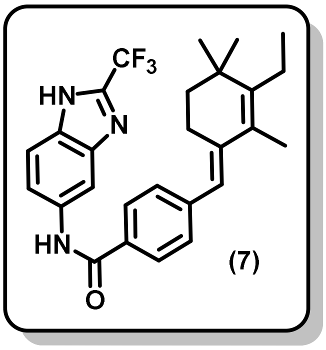

Pale yellow solid (Yield = 34%)

^{1}H NMR (400 MHz, DMSO-d_6) δ 8.30 (s, 1H), 7.85 (d, J = 8.1 Hz, 2H), 7.79 – 7.74 (m, 1H), 7.58 (d, J = 8.0 Hz, 2H), 7.23 (s, 1H), 6.59 (s, 1H), 2.67 (t, J = 6.3 Hz, 2H), 2.27 (q, J = 7.4 Hz, 2H), 1.95 (s, 3H), 1.53 (t, J = 6.5 Hz, 2H), 1.12 (d, J = 13.4 Hz, 9H). ^{13}C NMR (101 MHz, DMSO-d_6) δ 166.26, 149.44, 144.33, 142.71, 138.89, 130.83, 130.40, 129.89, 128.92, 127.17, 120.77, 119.01, 38.66, 36.04, 27.83, 24.36, 22.91, 15.43, 15.10. ^{19}F NMR (376.5 MHz, DMSO-d_6) δ -62.59. MS (ESI) calcd for $C_{27}H_{28}F_3N_3O$ 467.2184 found 467.3913[M+H].

(E)-4-(4-((3-ethyl-2,4,4-trimethylcyclohex-2-en-1-ylidene)methyl)benzamido)benzoic acid (8):

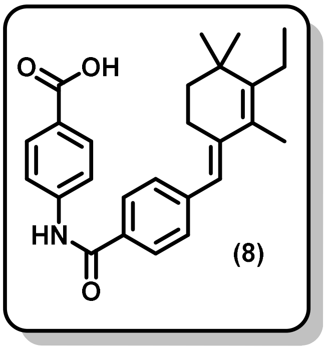

Pale yellow solid (Yield = 15%)

^{1}H NMR (400 MHz, DMSO-d_6) δ 7.72 – 7.68 (m, 4H), 7.35 (q, J = 8.2 Hz, 4H), 6.44 (s, 1H), 2.56 (t, J = 6.5 Hz, 2H), 2.19 (q, J = 7.6 Hz, 2H), 1.86 (s, 3H), 1.65 – 1.61 (m, 2H), 1.05 (d, J = 11.2

Hz, 9H). ^{13}C NMR (101 MHz, DMSO-d_6) δ 167.47, 148.07, 140.78, 132.20, 132.08, 129.27, 129.14, 127.35, 121.13, 67.89, 38.56, 30.27, 28.83, 23.72, 22.87, 14.36, 11.27. MS (ESI) calcd for $C_{26}H_{29}NO_3K$ 442.1785 found 442.4073 [M+H].

Procedure for the synthesis of (E)-4-((3-ethyl-2,4,4-trimethylcyclohex-2-en-1-ylidene)methyl)benzonitrile (9):

A clean oven-dried two-neck round bottom flask (RBF) was charged with salt (**3**) (1 equiv.) and 4-formyl benzonitrile (1 equiv.) (**10**) in DMF and stirred the reaction at room temperature until the formation of clear solution (10 minutes) Sodium tertbutoxide (3 equiv.) was added into the reaction mixture at 0 ^{0}C under nitrogen atmosphere by portion wise. The reaction mixture was stirred at room temperature for 24 hr. Progress of the reaction was monitored by TLC (50 % Hexane: 50 % ethyl acetate), In TLC polar spot was observed corresponding to aldehyde (**4**). After completion of the reaction, the reaction mixture was neutralized by 2N HCl. After neutralization, ethyl acetate was added to the reaction mixture; the combined organic layer was collected and dried with Na_2SO_4 and evaporated under reduced pressure. The crude material was purified by column chromatography. After purification (10% ethyl acetate: 30% hexane) yellow solid was observed.

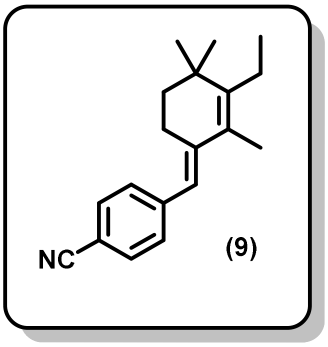

Yellow solid (Yield = 55 %)

^{1}H NMR (400 MHz, DMSO-d_6) δ 7.77 (d, J = 8.2 Hz, 2H), 7.47 (d, J = 8.2 Hz, 2H), 6.47 (s, 1H), 2.55 – 2.52 (m, 2H), 2.20 (d, J = 7.5 Hz, 2H), 1.86 (s, 3H), 1.48 – 1.38 (m, 2H), 1.05 – 1.00 (m, 9H).^{13}C NMR (101 MHz, DMSO-d_6) δ 149.57, 143.83, 142.79, 132.32, 130.32, 127.06, 120.55, 119.62, 108.41, 38.56, 36.00, 27.78, 24.24, 22.88, 15.38, 15.07. MS (ESI) calcd for $C_{19}H_{23}N$288.1728found 288.1687 [M+Na].

(E)-5-(4-((3-ethyl-2,4,4-trimethylcyclohex-2-en-1-ylidene)methyl)phenyl)-1H-tetrazole (10):

A clean oven-dried round bottom flask (RBF) was charged with nitrile (1) (1equiv), potassium carbonate (2) (3 equiv), and sodium azide(3) (3 equiv) in DMF and reflux at 100 under nitrogen atmosphere for 24 hr. Progress of the reaction was monitored by TLC (50 % Hexane: 50 % ethyl acetate), In TLC polar spot was observed corresponding to nitrile (1). After completion of the reaction, ice-cold water, and ethyl acetate were added and extracted thrice. The combined organic layer was collected and dried with Na_2SO_4 and evaporated under reduced pressure. The crude material was purified by column chromatography (100% ethyl acetate: 0 % hexane) after purification cream solid was observed.

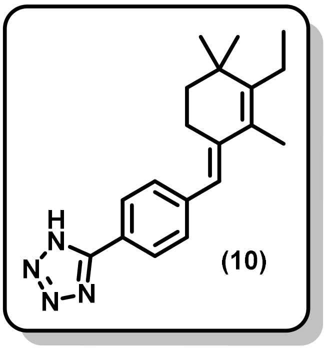

Cream solid (Yield = 62 %)

^{1}H NMR (400 MHz, DMSO) δ 8.00 (d, *J* = 7.9 Hz, 2H), 7.51 (d, *J* = 7.9 Hz, 2H), 6.49 (s, 1H), 2.60 (s, 2H), 2.21 (d, *J* = 7.4 Hz, 2H), 1.88 (s, 3H), 1.47 (s, 2H), 1.06-1.01 (m,9H).^{13}C NMR (101 MHz, DMSO-*d6*) δ ^{13}C NMR (101 MHz, DMSO) δ 148.67, 141.73, 141.57, 130.41, 127.10, 120.93, 38.68, 35.97, 27.86, 24.31, 22.85, 15.40, 15.13. HRMS (ESI) calcd for $C_{19}H_{25}N_4$ 309.2001 found 309.1399 $[M+H]^+$.

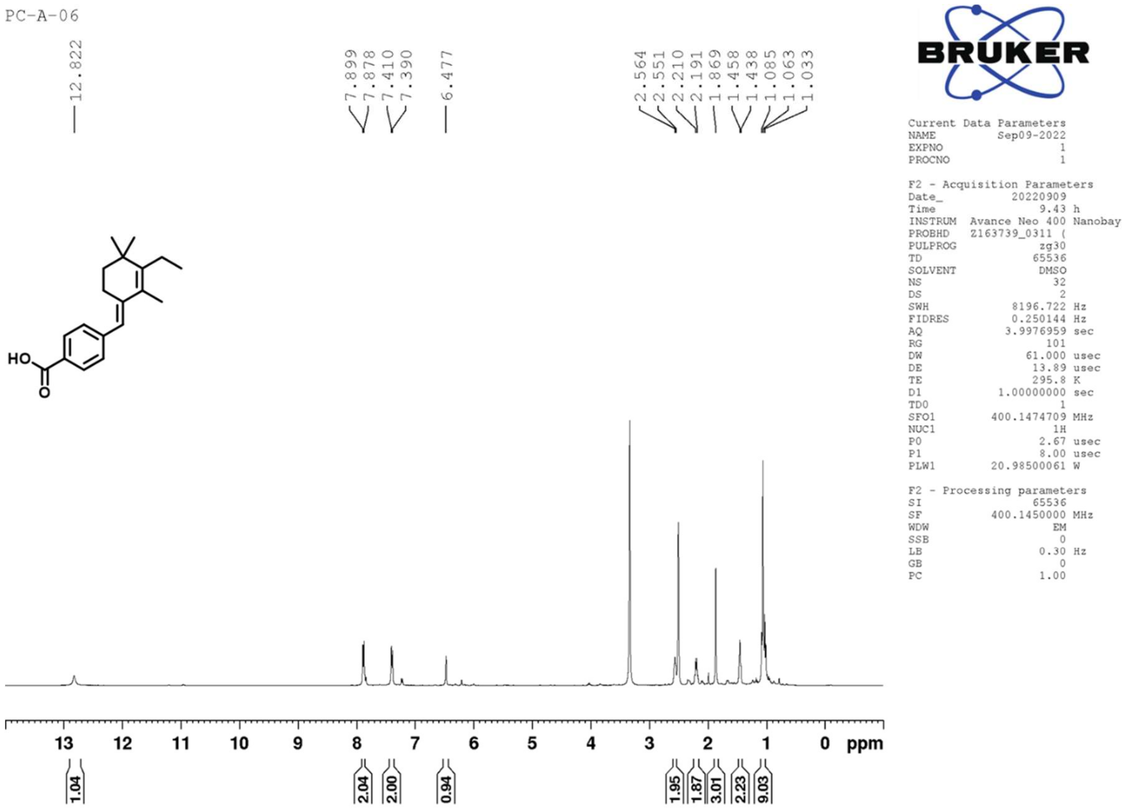

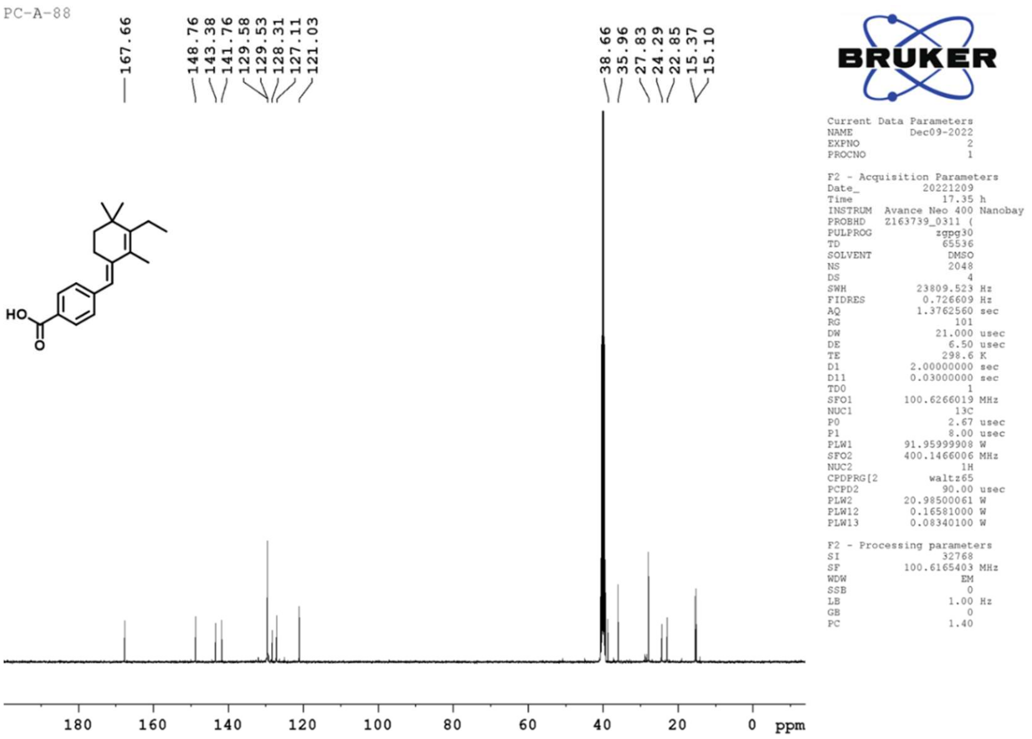

Figure S1. ^{1}H & ^{13}C NMR data of compound **4**

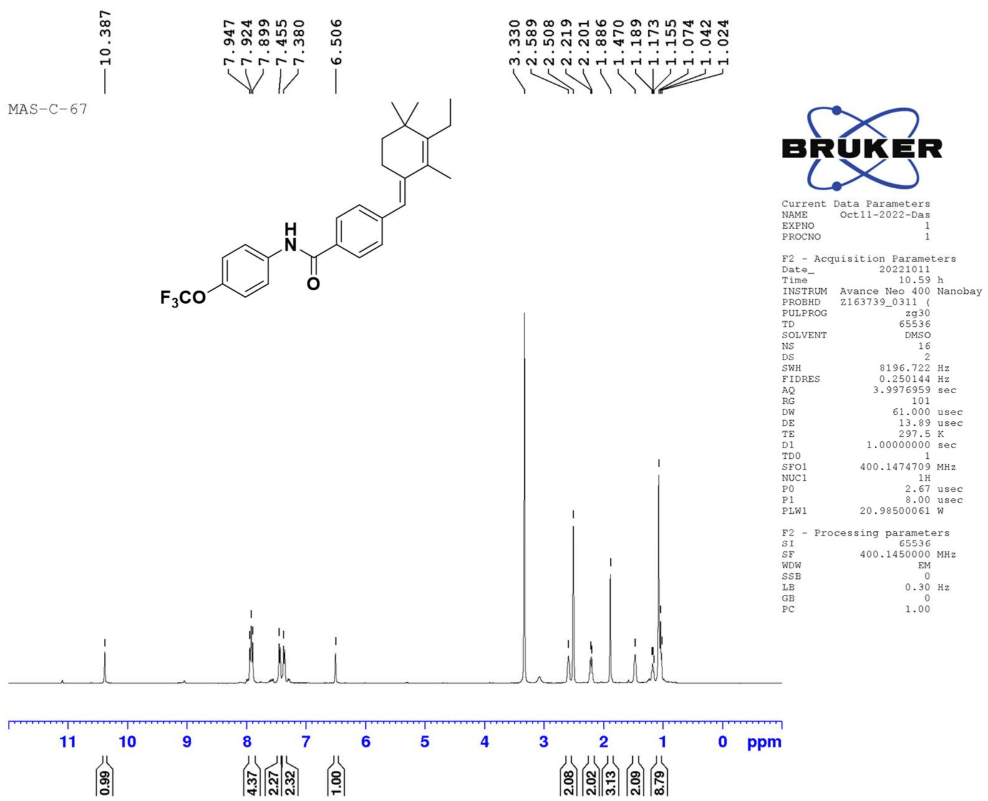
MAS-C-67
10.387
7.947
7.924
7.899
7.455
7.380
6.506
3.330
2.589
2.508
2.219
2.201
1.886
1.470
1.189
1.173
1.155
1.074
1.042
1.024
H
N
O
F3CO
BRUKER
Current Data Parameters
NAME Oct11-2022-Das
EXPNO 1
PROCNO 1
F2 - Acquisition Parameters
Date_ 20221011
Time 10.59 h
INSTRUM Avance Neo 400 Nanobay
PROBHD Z163739_0311 (
PULPROG zg30
TD 65536
SOLVENT DMSO
NS 16
DS 2
SWH 8196.722 Hz
FIDRES 0.250144 Hz
AQ 3.9976959 sec
RG 101
DW 61.000 usec
DE 13.89 usec
TE 297.5 K
D1 1.00000000 sec
TD0 1
SFO1 400.1474709 MHz
NUC1 1H
P0 2.67 usec
P1 8.00 usec
PLW1 20.98500061 W
F2 - Processing parameters
SI 65536
SF 400.1450000 MHz
WDW EM
SSB 0
LB 0.30 Hz
GB 0
PC 1.00
11
10
9
8
7
6
5
4
3
2
1
0
ppm
0.99
4.37
2.27
2.32
1.00
2.08
2.02
3.13
2.09
8.79

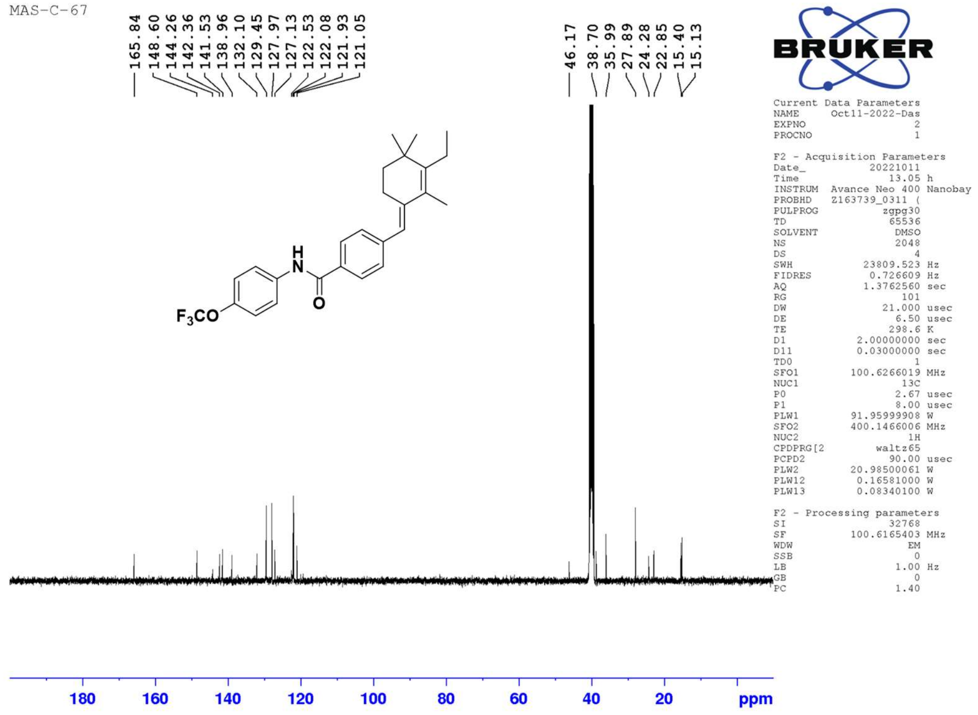
MAS-C-67
165.84
148.60
144.26
142.36
141.53
138.96
132.10
129.45
127.97
127.13
122.53
122.08
121.93
121.05
46.17
38.70
35.99
27.89
24.28
22.85
15.40
15.13
H
N
O
F3CO
BRUKER
Current Data Parameters
NAME Oct11-2022-Das
EXPNO 2
PROCNO 1
F2 - Acquisition Parameters
Date_ 20221011
Time 13.05 h
INSTRUM Avance Neo 400 Nanobay
PROBHD Z163739_0311 (
PULPROG zgpg30
TD 65536
SOLVENT DMSO
NS 2048
DS 4
SWH 23809.523 Hz
FIDRES 0.726609 Hz
AQ 1.3762560 sec
RG 101
DW 21.000 usec
DE 6.50 usec
TE 298.6 K
D1 2.00000000 sec
D11 0.03000000 sec
TD0 1
SFO1 100.6266019 MHz
NUC1 13C
P0 2.67 usec
P1 8.00 usec
PLW1 91.95999908 W
SFO2 400.1466006 MHz
NUC2 1H
CPDPRG[2 waltz65
PCPD2 90.00 usec
PLW2 20.98500061 W
PLW12 0.16581000 W
PLW13 0.08340100 W
F2 - Processing parameters
SI 32768
SF 100.6165403 MHz
WDW EM
SSB 0
LB 1.00 Hz
GB 0
PC 1.40
180
160
140
120
100
80
60
40
20
ppm

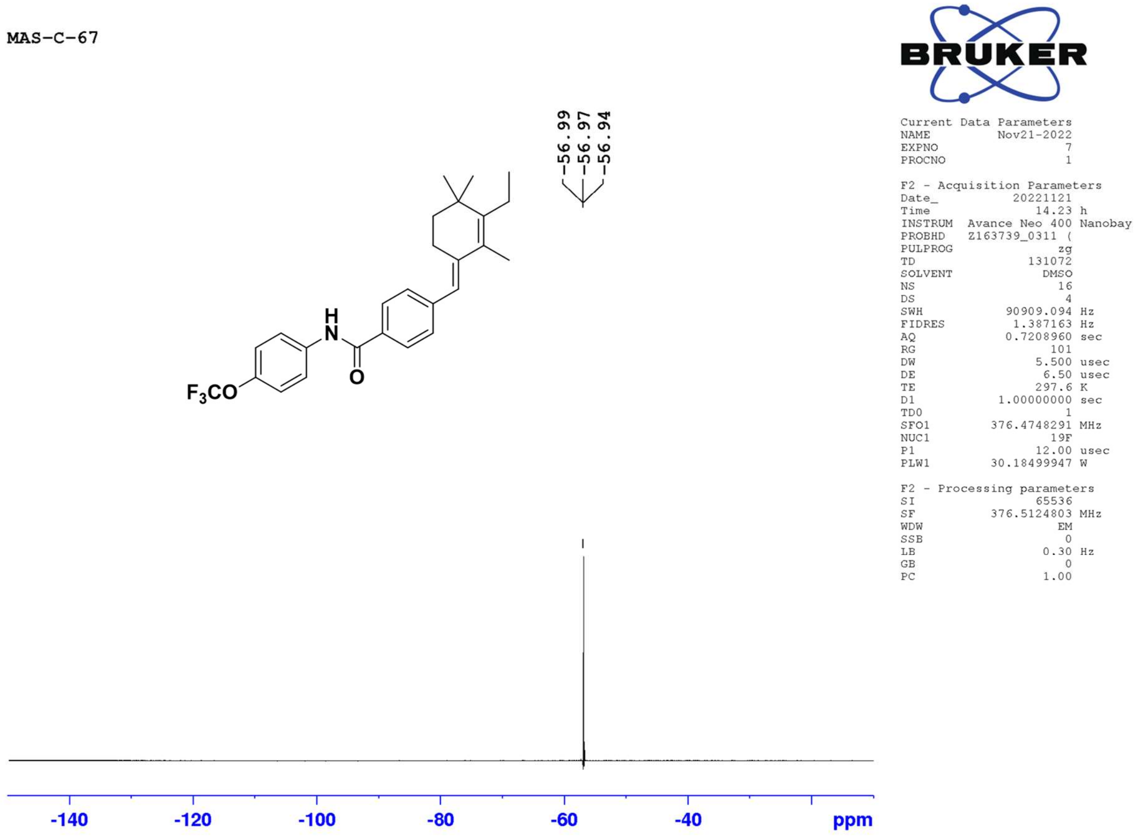

Figure S2. ^{1}H, ^{13}C & ^{19}F NMR data of compound **5**

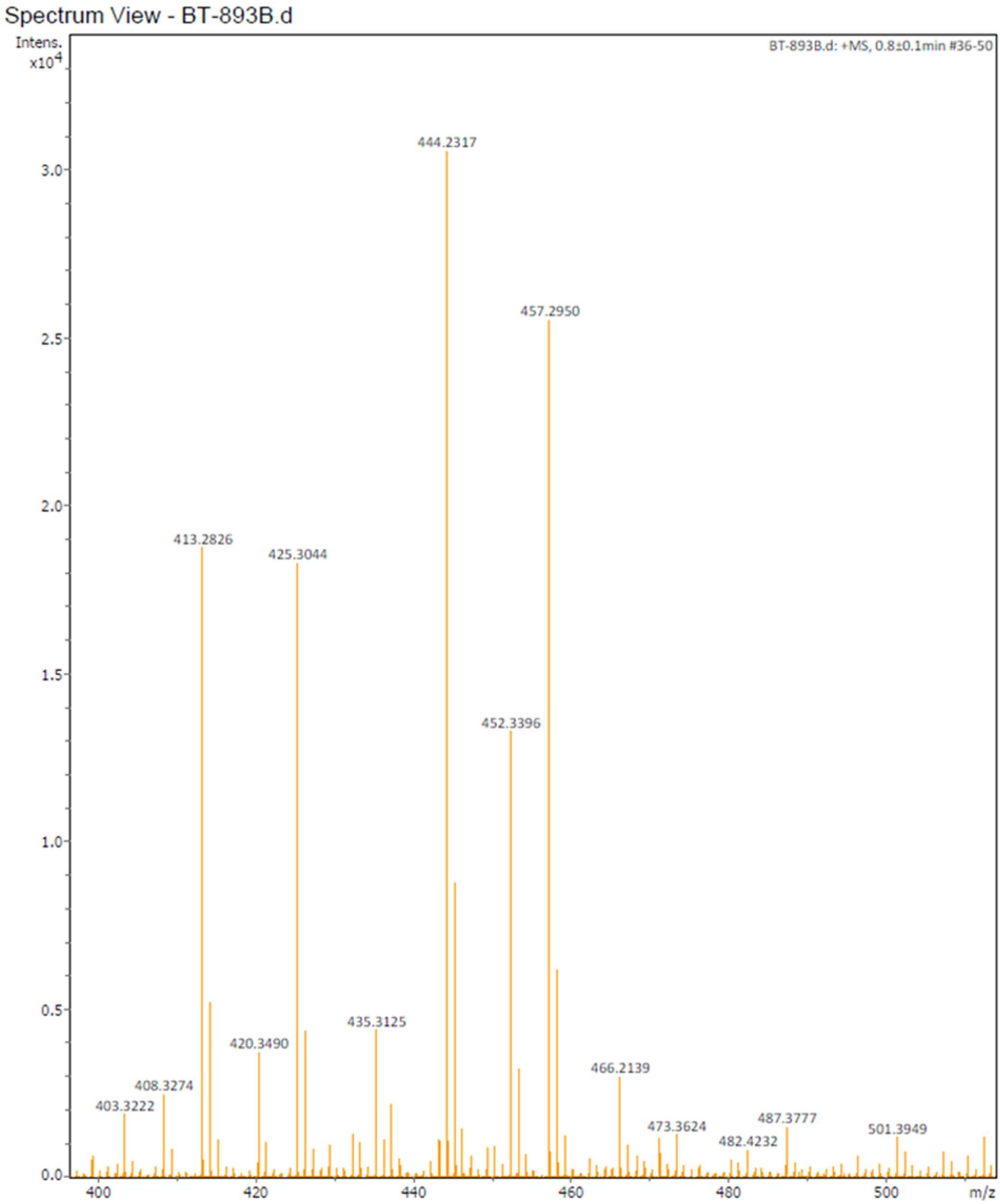

Figure S3. LCMS data of compound **5**

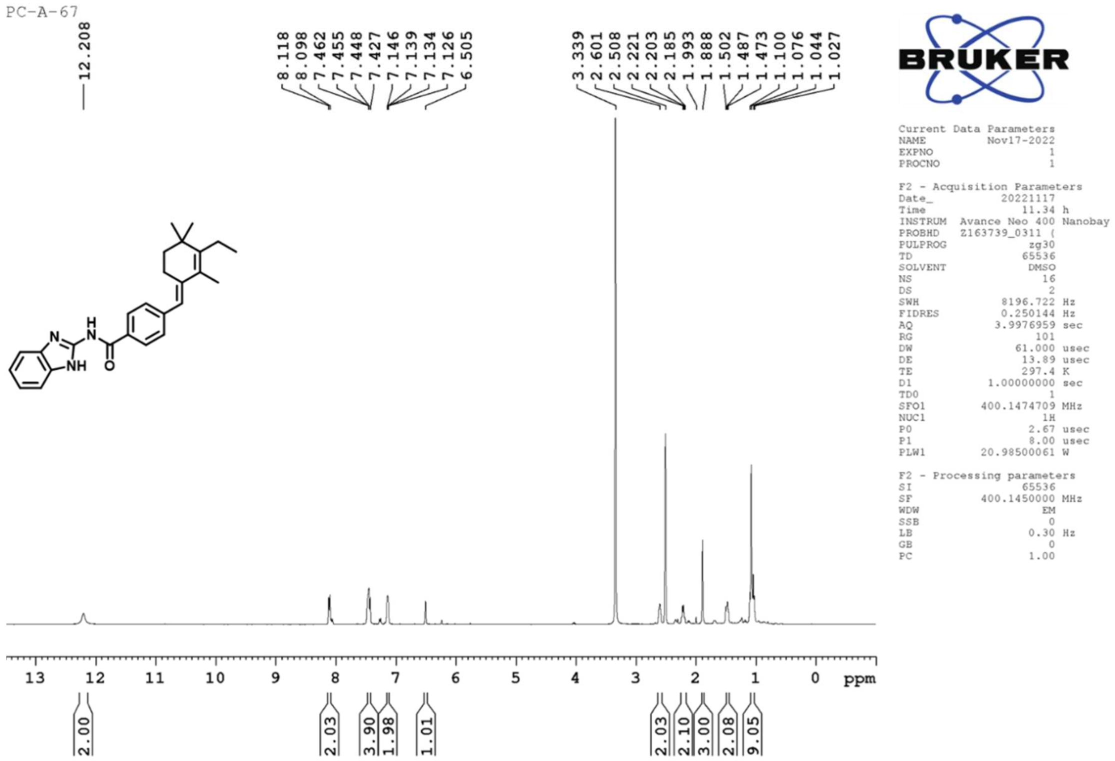

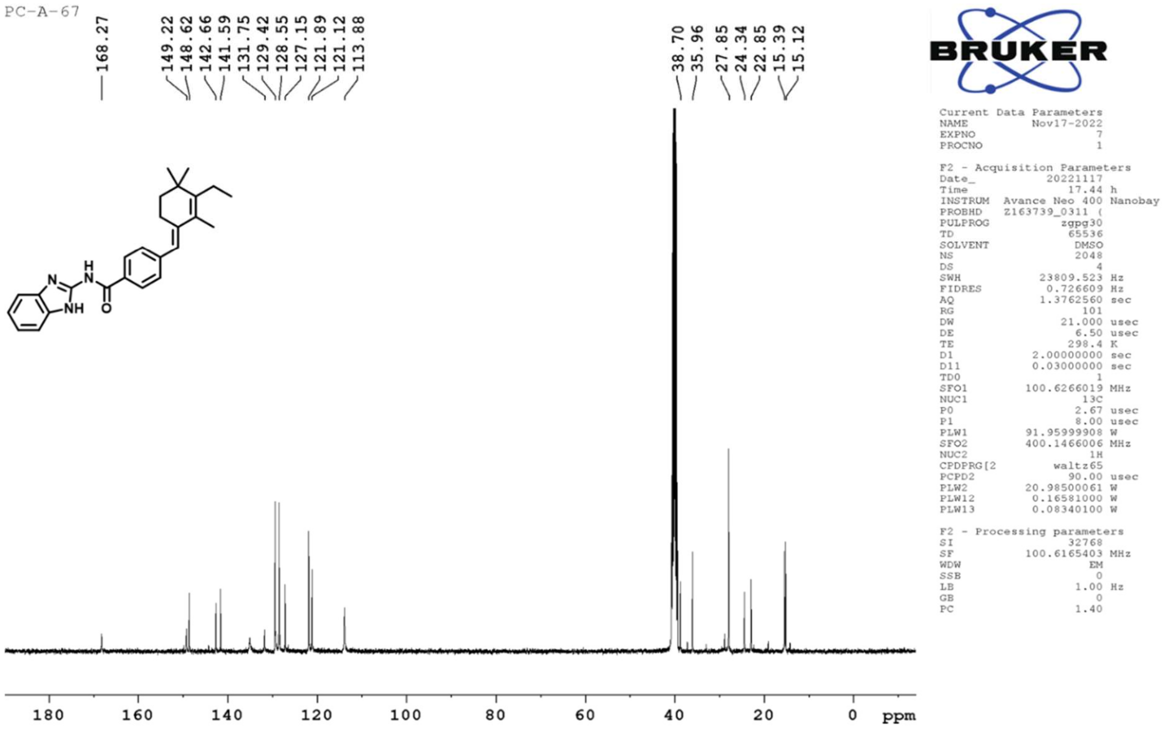

Figure S4. ^{1}H & ^{13}C NMR data of compound **6**

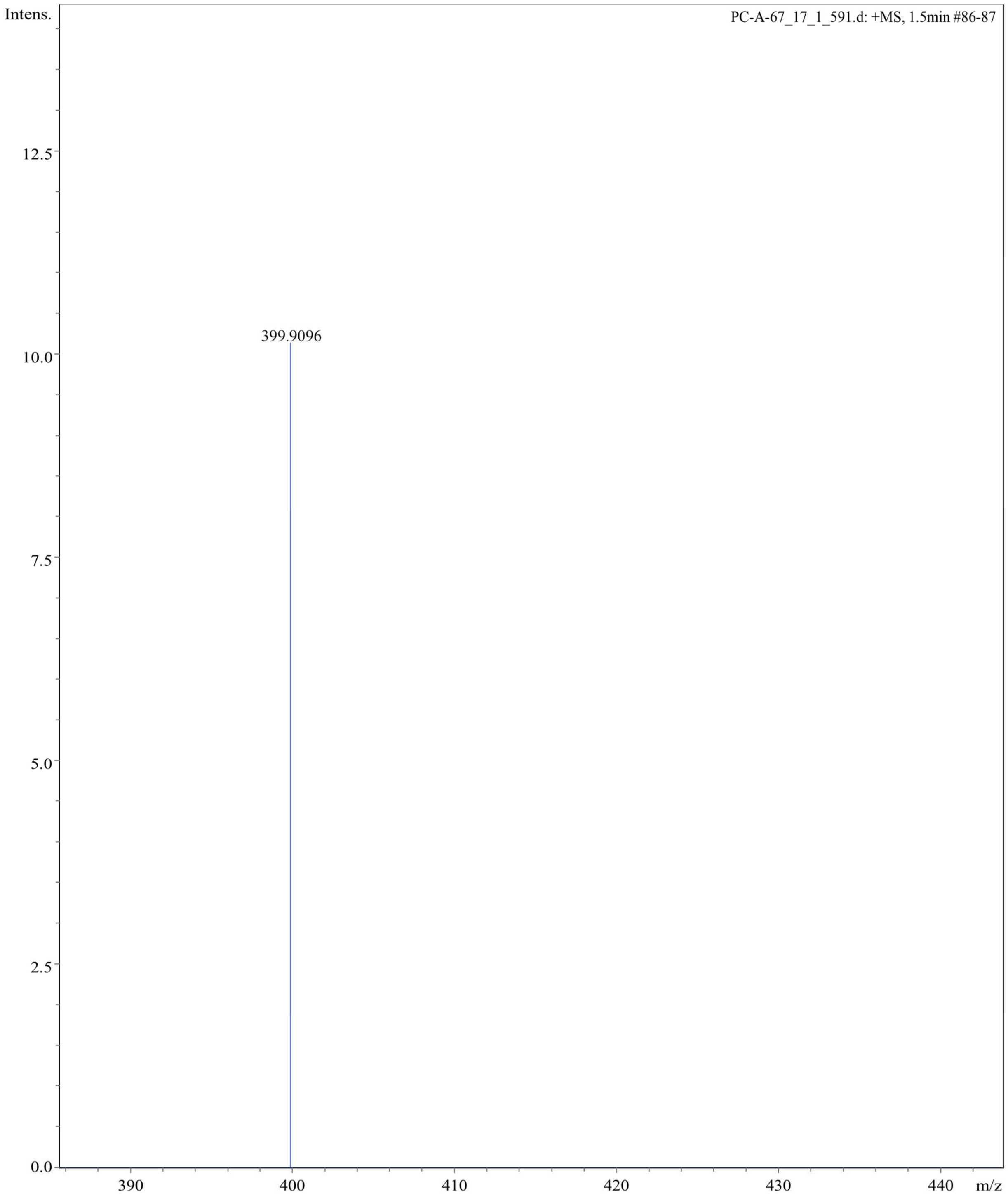

Bruker Compass DataAnalysis 5.2 printed: 6/26/2023 1:46:11 PM by: demo Page 1 of 1

Figure S5. LCMS data of compound **6**

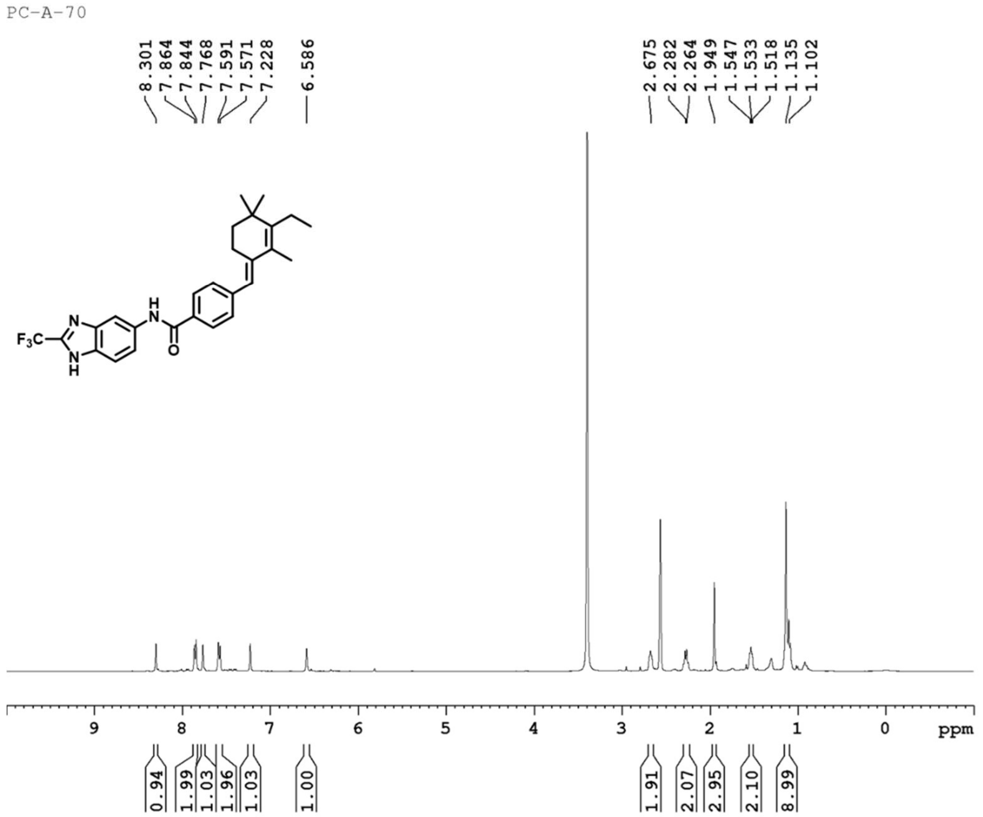

Current Data Parameters
NAME Nov18-2022
EXPNO 4
PROCNO 1

F2 - Acquisition Parameters
Date_ 20221118
Time 12.13 h
INSTRUM Avance Neo 400 Nanobay
PROBHD Z163739_0311 (
PULPROG zg30
TD 65536
SOLVENT DMSO
NS 64
DS 2
SWH 8196.722 Hz
FIDRES 0.250144 Hz
AQ 3.9976959 sec
RG 101
DW 61.000 usec
DE 13.89 usec
TE 297.5 K
D1 1.00000000 sec
TD0 1
SFO1 400.1474709 MHz
NUC1 1H
P0 2.67 usec
P1 8.00 usec
PLW1 20.98500061 W

F2 - Processing parameters
SI 65536
SF 400.1449778 MHz
WDW EM
SSB 0
LB 0.30 Hz
GB 0
PC 1.00

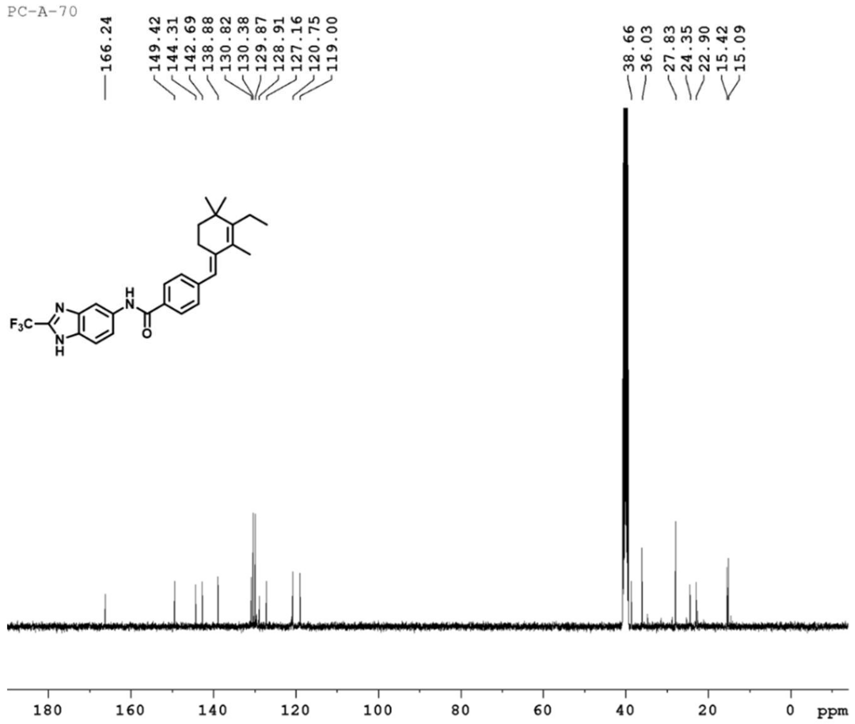

Current Data Parameters
NAME Nov18-2022
EXPNO 8
PROCNO 1

F2 - Acquisition Parameters
Date_ 20221118
Time 20.13 h
INSTRUM Avance Neo 400 Nanobay
PROBHD Z163739_0311 (
PULPROG zgpg30
TD 65536
SOLVENT DMSO
NS 3072
DS 4
SWH 23809.523 Hz
FIDRES 0.726609 Hz
AQ 1.3762560 sec
RG 101
DW 21.000 usec
DE 6.50 usec
TE 298.6 K
D1 2.00000000 sec
D11 0.03000000 sec
TD0 1
SFO1 100.6266019 MHz
NUC1 13C
P0 2.67 usec
P1 8.00 usec
PLW1 91.95999908 W
SFO2 400.1466006 MHz
NUC2 1H
CPDPRG[2 waltz65
PCPD2 90.00 usec
PLW2 20.98500061 W
PLW12 0.16581000 W
PLW13 0.08340100 W

F2 - Processing parameters
SI 32768
SF 100.6165403 MHz
WDW EM
SSB 0
LB 1.00 Hz
GB 0
PC 1.40

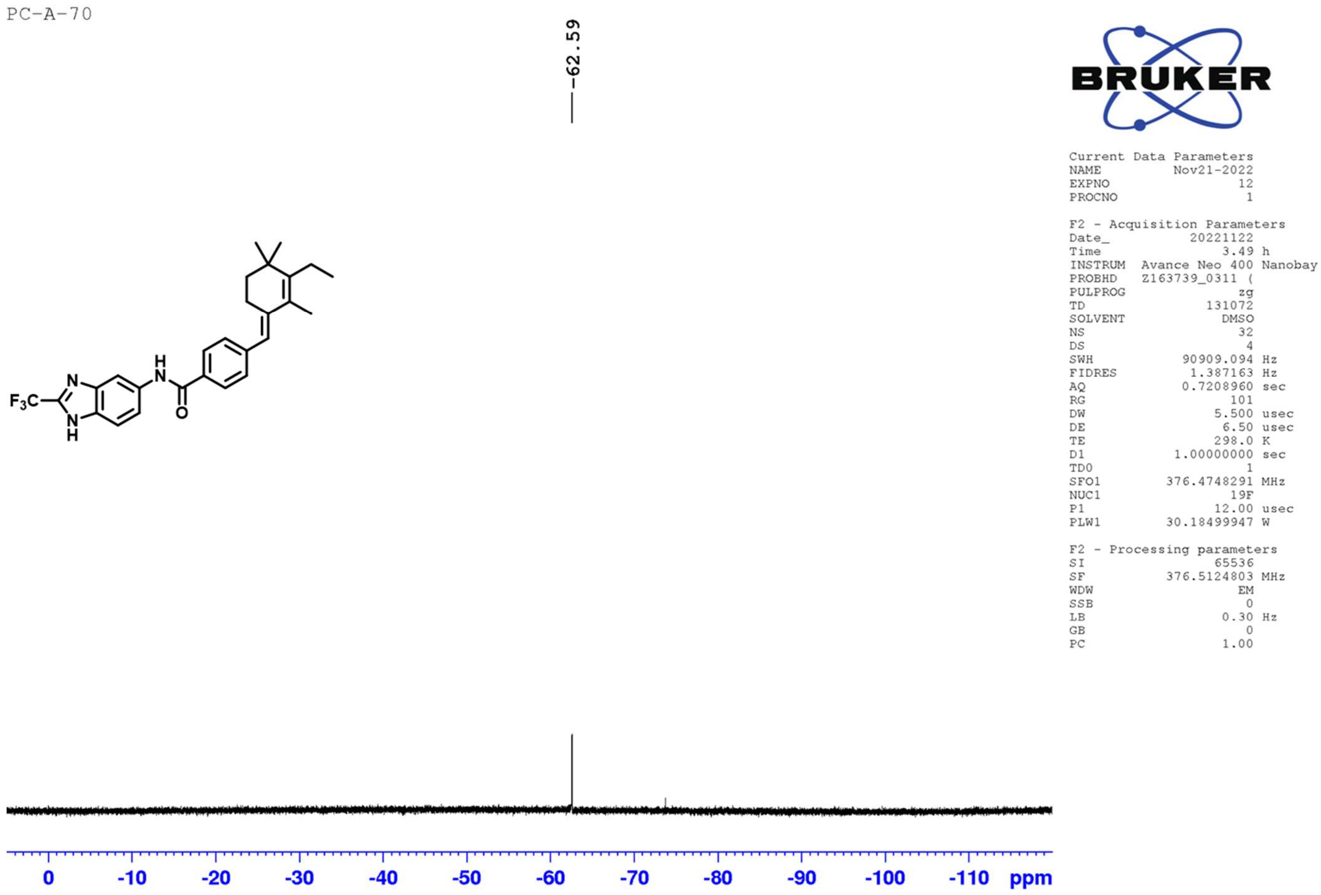

Figure S6. ^{1}H, ^{13}C & ^{19}F NMR data of compound **7**

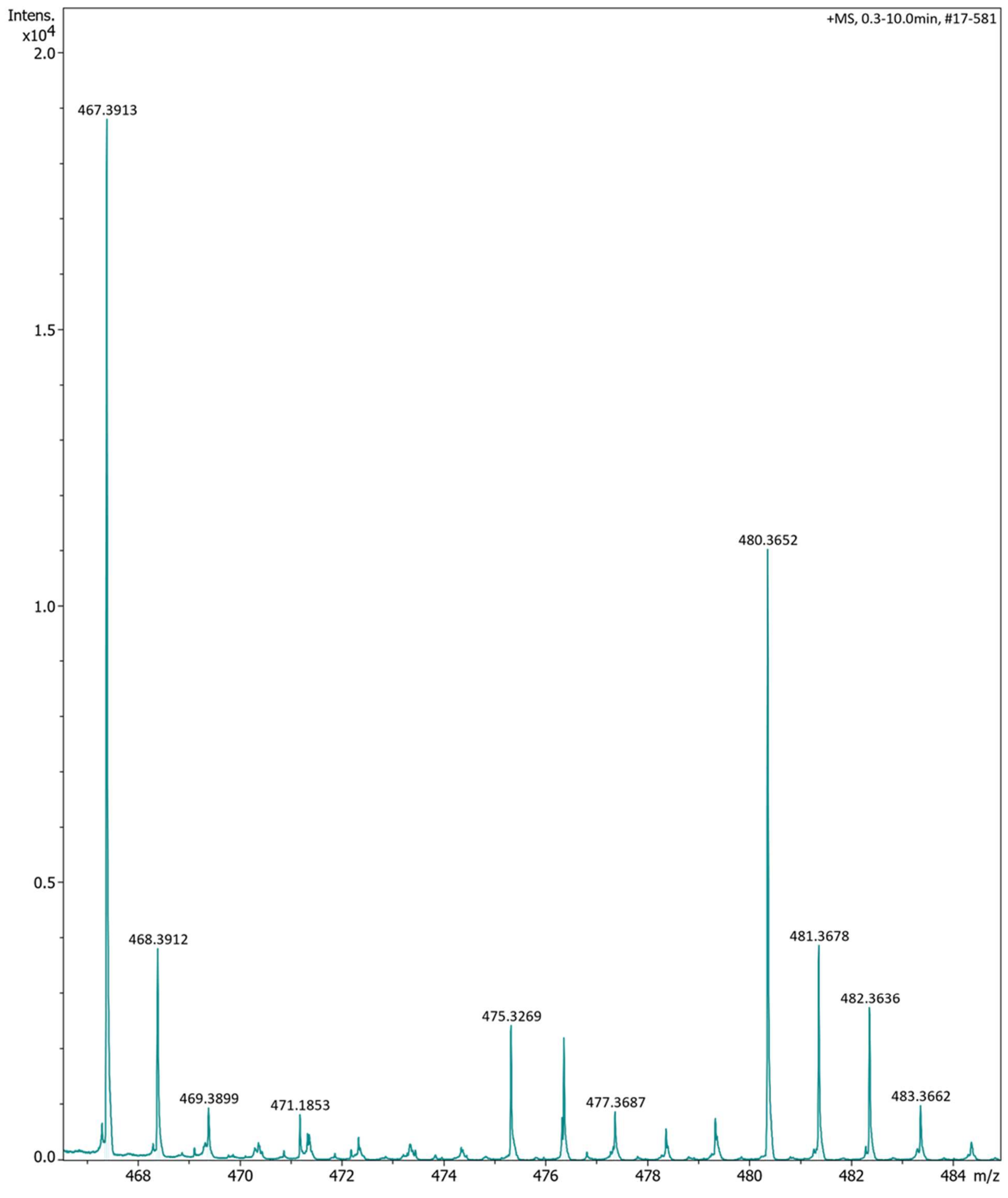

Bruker Compass DataAnalysis 6.0 printed: 5/3/2024 11:40:50 AM by: demo Page 1 of 1

Figure S7. LCMS data of compound **7**

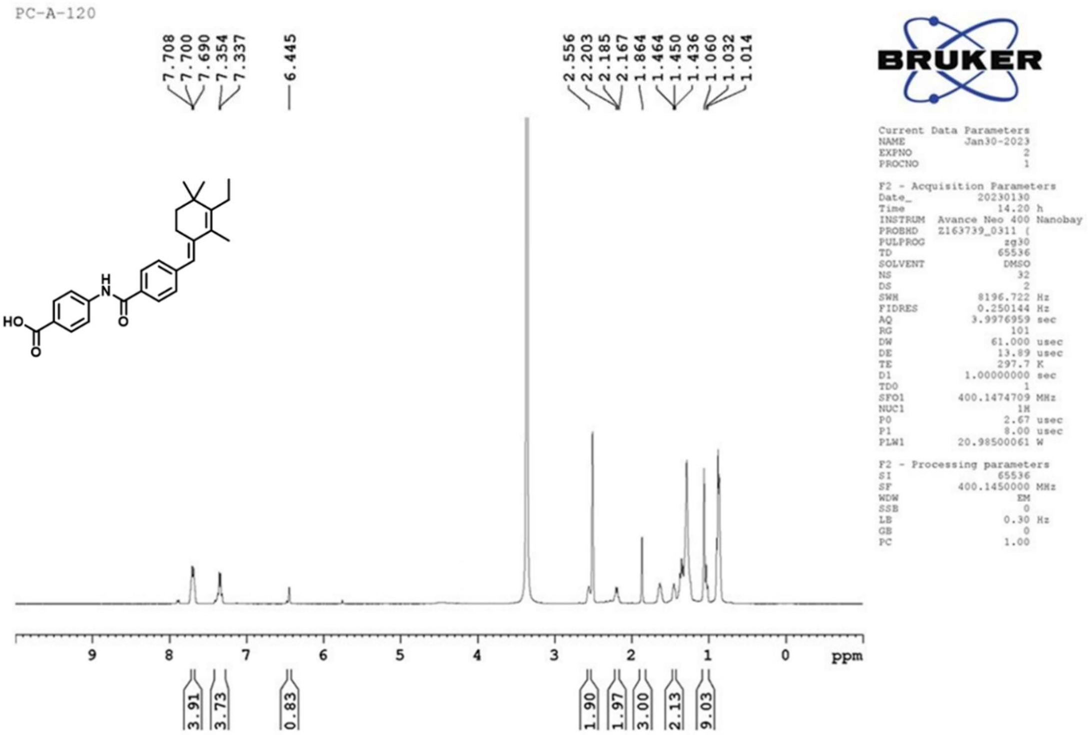

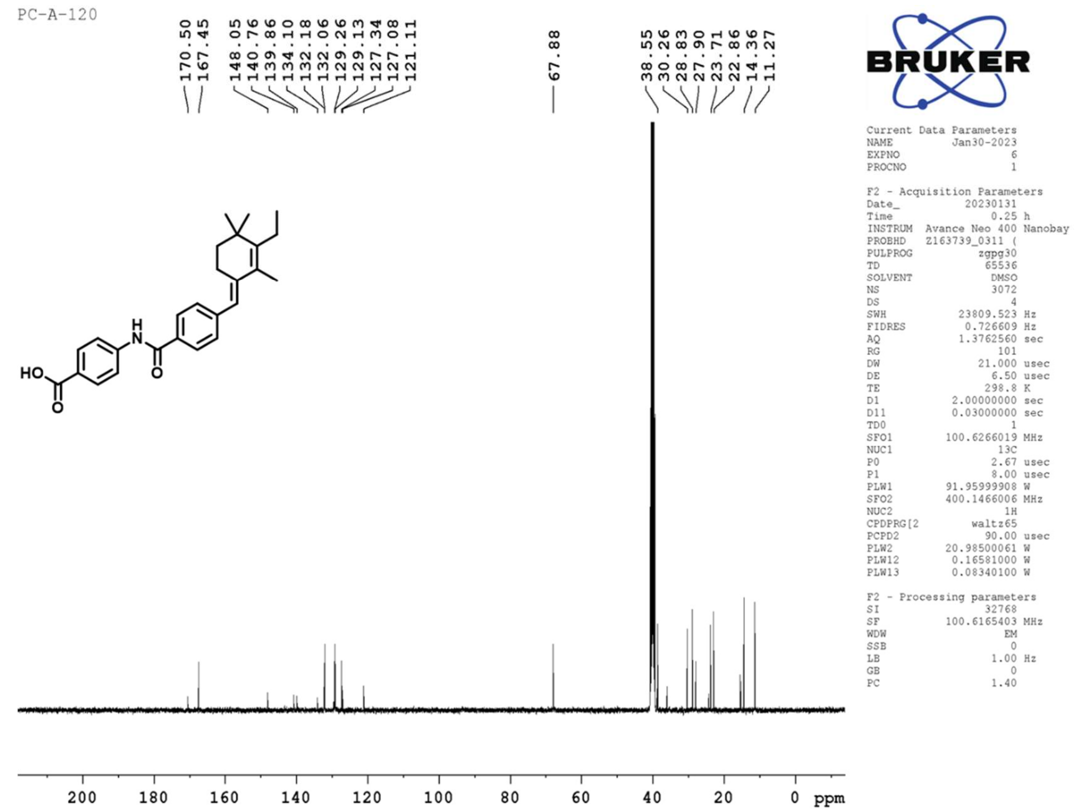

Figure S8. ^{1}H & ^{13}C NMR data of compound **8**

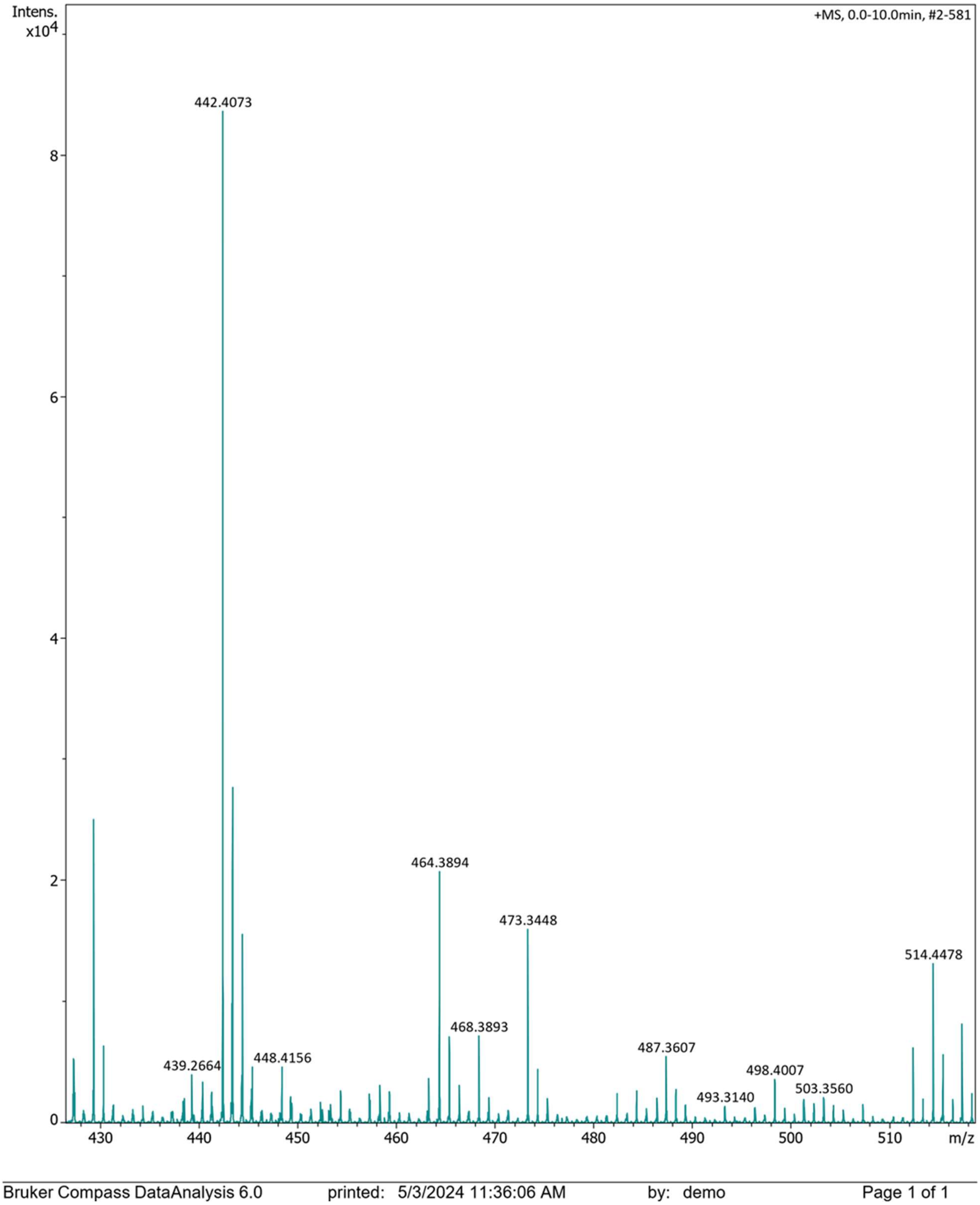

Figure S9. LCMS data of compound **8**

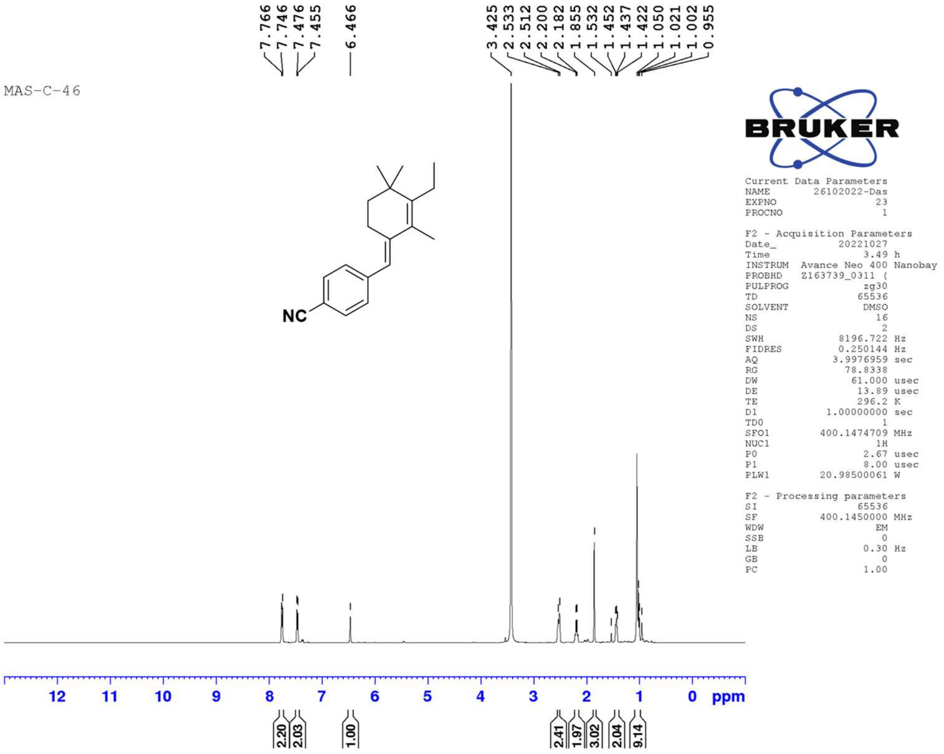

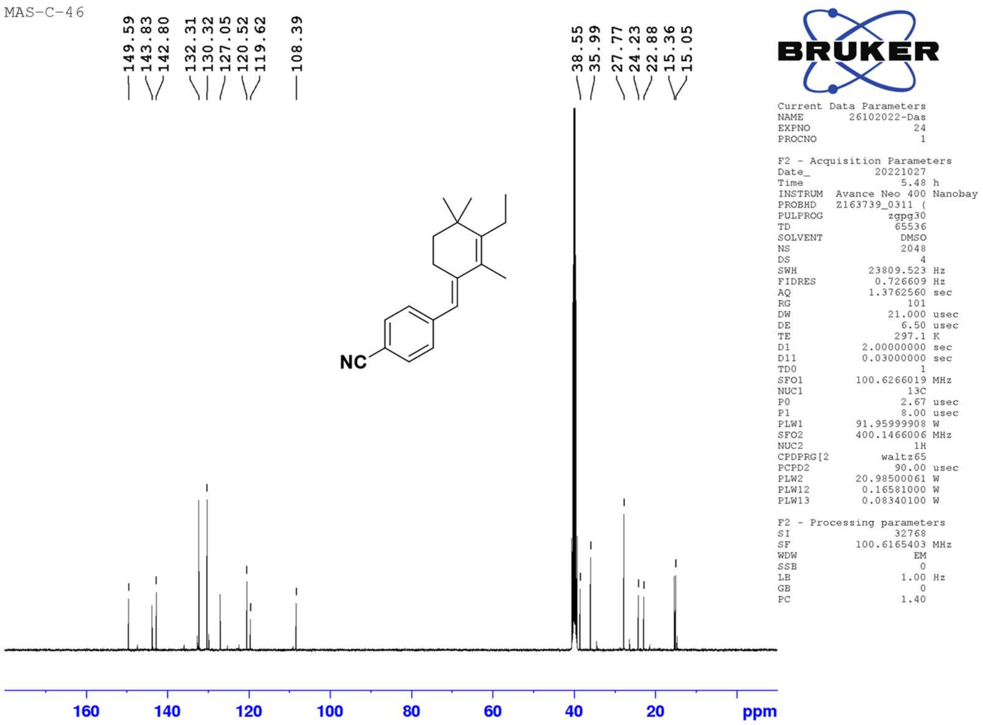

Figure S10. ^{1}H & ^{13}C NMR data of compound **9**

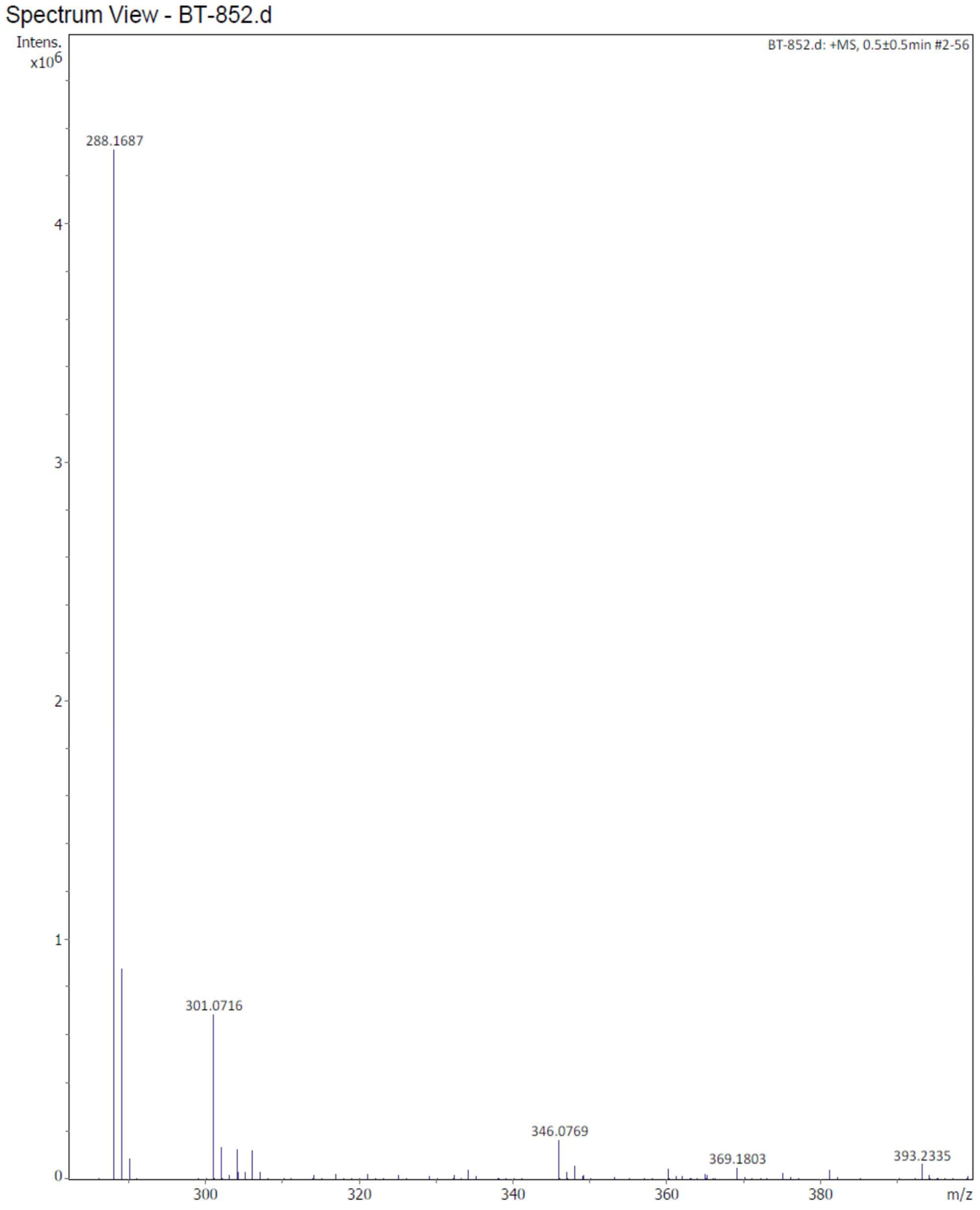

Figure S11. LCMS data of compound **9**

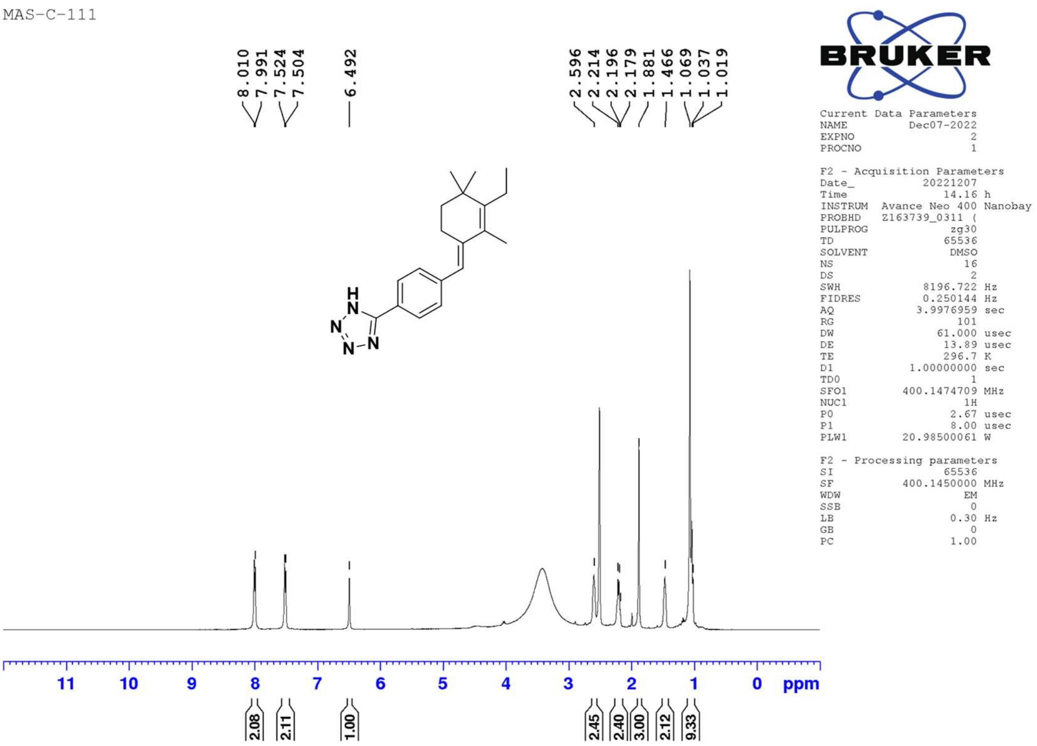

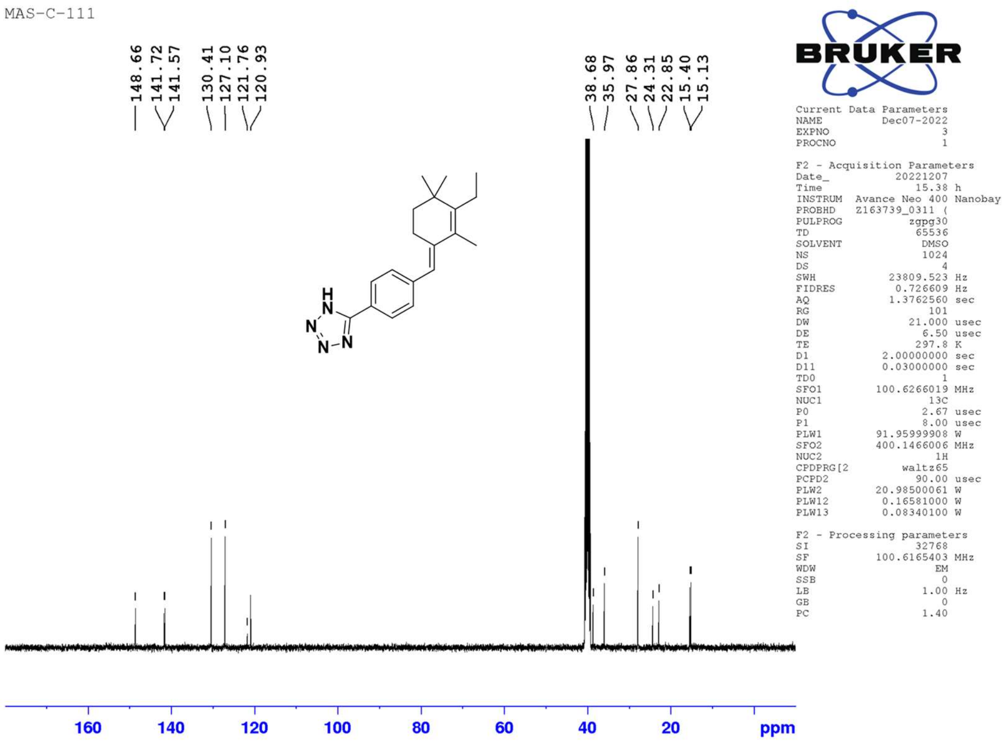

Figure S12. ^{1}H & ^{13}C NMR data of compound **10**

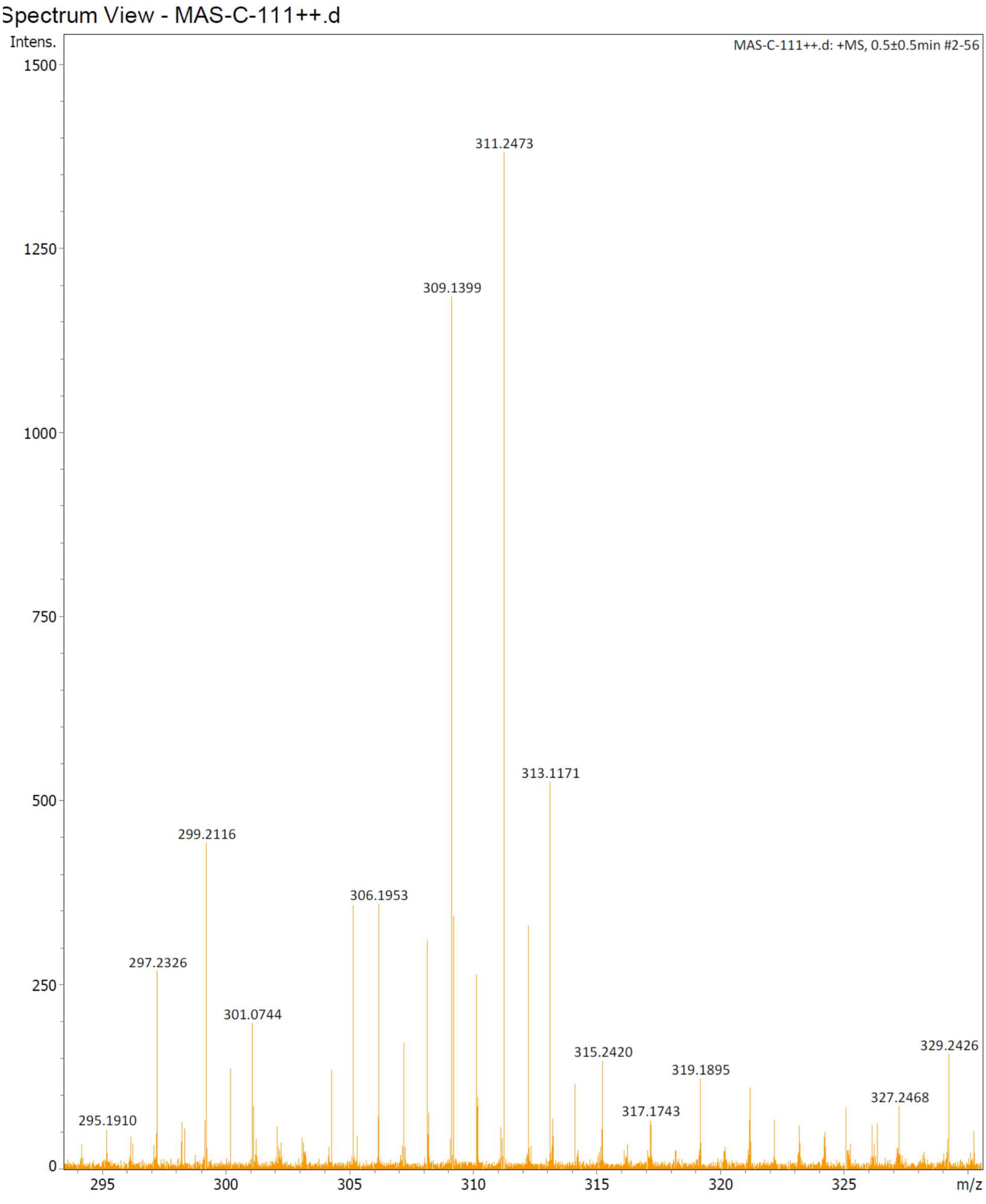

Figure S13. LCMS data of compound **10**

5.0 Molecular Docking

The Cambridge Crystallographic Data Centre (CCDC) software GOLD was employed to perform docking studies to support the interaction and preferred binding model of the compound BL6. The active sites of the Human MetAP2 protein (PDB ID: *1b6a*) and Protozoal MetAP2 (PDB ID: *3fmq*) were utilized for docking BL6. Discovery Studio Visualizer was used to generate 2D and 3D images of the docking and binding interactions. The crystallized structures of the proteins were derived, and the docking of the BL6 ligand was carried out using a series of progressively refined protocols available in GOLD. Initially, the virtual screening protocol was used to dock BL6 to both the entire surface of MetAP2 and specifically to the ligand binding site. Next, a rigid receptor protocol was employed to refine the docked conformations at the binding site. Finally, an induced fit protocol was used to allow for protein flexibility at the cleft binding site, further refining the binding affinity.

BL6 exhibits a variety of molecular interactions with the amino acid residues lining the binding cavity and the di-metal co-factor CO2+ of Human METAP2. One significant interaction is the hydrogen bond formed with the residue Tyr321, which plays a crucial role in stabilizing the ligand-protein complex and enhancing binding affinity. Additionally, BL6 engages in a pi-pi T-shaped interaction with His216, which involves the aromatic rings of both BL6 and the histidine residue, further contributing to the stability of the complex. Van-der-Waals interactions with the CO2+ co-factor and other residues in the binding cavity add to the binding affinity by providing additional non-covalent stabilization. Alkyl interactions with various residues also play a role in this stabilization process.

In contrast, BL6 exhibits stronger interactions with Protozoal METAP2. It forms hydrogen bonds with His572, His562, and Ser576, which are critical for strong binding and stability. Van-der-Waals interactions with Pro452, His615, Asp495, and the co-factor Fe(III) ion further enhance the binding affinity by providing a network of weak but collectively significant stabilizing forces. Additionally, BL6 participates in Pi-Sigma and Pi-Pi interactions with various residues, which contribute to the overall binding affinity observed with Protozoal METAP2. These interactions illustrate the intricate ways in which BL6 can bind effectively to both Human and Protozoal METAP2, highlighting the diverse types of molecular interactions involved.

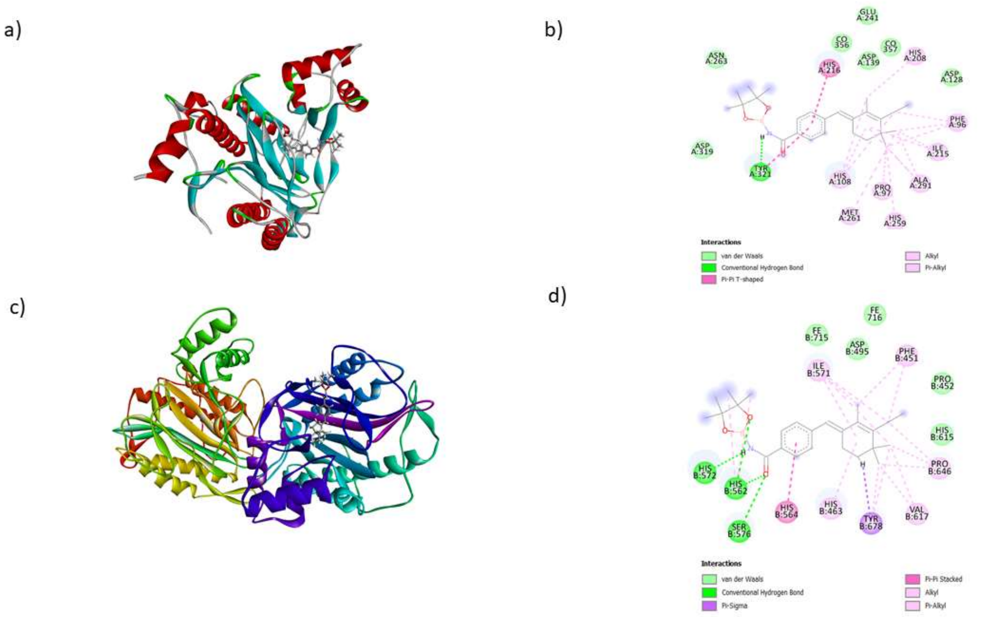

Figure S14 :A)3D representation of HuMetAP2 (PDB ID:*1b6a*) with BL6. **B)** Ligand interaction 2D mapping of HuMetAP2/BL-6. **C)** 3D representation of EcMetAP2 (PDB ID:*3fmq*) with BL6. **D)** Ligand interaction 2D mapping of EcMetAP2/BL6.

Figure S15. Original uncropped immunoblot images

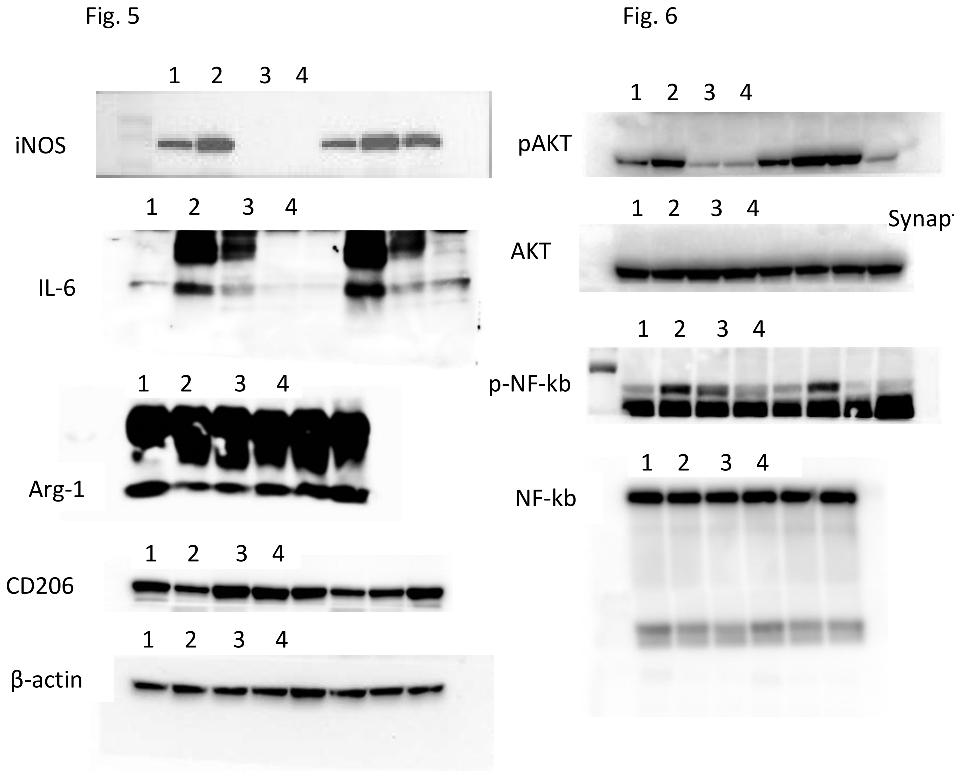

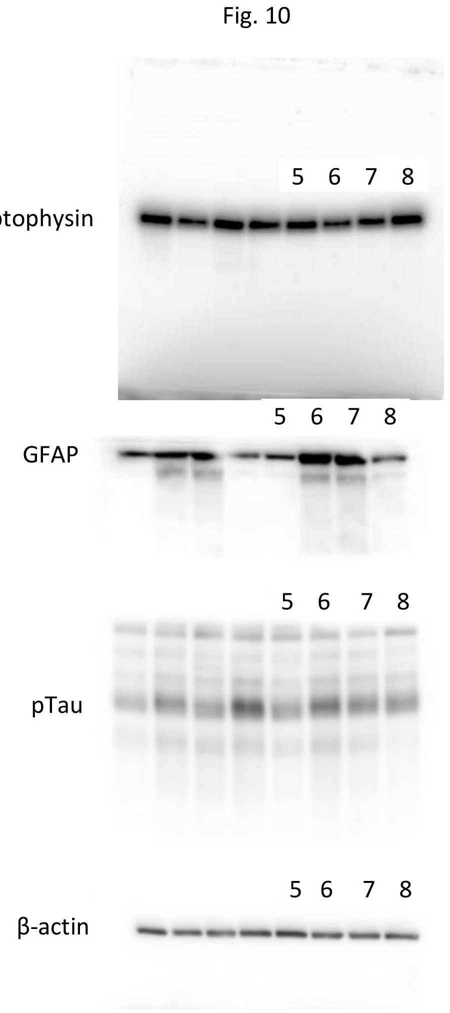

Fig. S15. Original uncropped immunoblot images

1. Control
2. LPS
3. LPS+BL6
4. BL6
5. Control
6. STZ
7. STZ+BL6
8. BL6
